# Egg-Derived Proteins as Functional Food Ingredients: Modification Strategies, Bioactive Peptides, Bioavailability, and Techno-Functional Applications

**DOI:** 10.3390/foods15213817

**Published:** 2026-10-26

**Authors:** Marius Giorgi Usturoi, Roxana Nicoleta Ratu, Alexandru Usturoi

**Affiliations:** 1 Department of Management of Animal Resources and Technologies, Faculty of Food and Animal Sciences, “Ion Ionescu de la Brad” Iasi University of Life Sciences, 3 Mihail Sadoveanu Alley, 700489 Iasi, Romania; marius.usturoi@iuls.ro; 2 Department of Food Technologies, Faculty of Agriculture, “Ion Ionescu de la Brad” Iasi University of Life Sciences, 3 Mihail Sadoveanu Alley, 700489 Iasi, Romania; roxana.ratu@iuls.ro; 3 Department of Control, Expertise and Services, Faculty of Food and Animal Sciences, “Ion Ionescu de la Brad” Iasi University of Life Sciences, 3 Mihail Sadoveanu Alley, 700489 Iasi, Romania

**Keywords:** egg-derived proteins, protein modification, bioactive peptides, bioavailability, techno-functional properties, allergenicity

## Abstract

Egg-derived proteins are increasingly investigated as multifunctional ingredients for the development of foods that combine nutritional value, technological performance and potential biological functionality. This structured narrative review critically synthesizes the recent literature on egg white and egg yolk proteins as precursors of techno-functional and bioactive food ingredients. The review focuses on enzymatic hydrolysis, fermentation-assisted modification, physical technologies and chemical or biochemical strategies, and links these interventions with molecular rearrangements, peptide release, digestibility, bioavailability, allergenicity and performance in food matrices. Current evidence indicates that controlled modification can improve solubility, foaming, emulsification, gelation, mineral-binding capacity and the release of peptides with antioxidant, anti-inflammatory, antihypertensive, antimicrobial and immunomodulatory potential. However, functional improvement is strongly process- and matrix-dependent, while most evidence for the reported biological activities remains preclinical, being derived mainly from in vitro assays and animal studies, and does not establish physiological efficacy in humans. Translation into functional foods remains limited by incomplete peptide standardization, scarce human validation, possible persistence or alteration of allergenicity, sensory defects, clean-label constraints, processing reproducibility and scalability. Overall, egg-derived proteins represent promising platforms for next-generation functional foods, provided that future research integrates molecular characterization with digestion and absorption studies, safety assessment, sensory evaluation, pilot-scale processing and validation in real food systems.

## 1. Introduction

Egg-derived proteins combine high nutritional quality with diverse technological functions. Egg white contains ovalbumin, ovotransferrin, ovomucoid, ovomucin, lysozyme and other proteins involved in foaming, gelation, coagulation, film formation and emulsion stabilization. Egg yolk proteins, lipoproteins and phosphoproteins contribute mainly to emulsification, interfacial stabilization, lipid structuring, mineral binding and texture development [1,2,3,4,5]. These properties support the established use of egg ingredients in bakery products, confectionery, sauces, dressings, desserts, meat products and other structured or emulsified foods. Egg proteins are also being investigated as components of gels, particles, emulsions, nanocomplexes and oral delivery systems [6,7,8,9]. Their relevance therefore extends beyond amino acid supply to include food structuring, stabilization and bioactive-compound delivery.

Protein-related factors can influence the quality of poultry-derived foods throughout production and processing. Dietary crude-protein strategies have been evaluated in slow-growing broilers in relation to production performance and meat quality [10]; however, this review is restricted to the composition, modification and functional-food applications of egg-derived proteins. Processing and storage are particularly relevant to egg ingredients. Packaging and storage conditions can modify the composition, stability and technological quality of powdered egg products [11,12], whereas pH, ionic strength, temperature, shear, protein concentration and interactions with lipids, polysaccharides or phenolic compounds influence protein conformation, aggregation and interfacial behavior [13,14]. Consequently, the performance of an egg-derived ingredient cannot be inferred solely from tests conducted using freshly prepared or purified protein dispersions.

Controlled modification is used to adapt egg proteins to specific technological and nutritional requirements. Enzymatic hydrolysis, fermentation, pH-shifting, ultrasound, cold plasma and high-pressure processing can alter secondary and tertiary structure, particle size, surface charge, hydrophobicity, aggregation and interfacial adsorption [15,16,17,18,19,20]. Maillard-type conjugation, phosphorylation, succinylation and other chemical or biochemical treatments provide additional control over hydration, electrostatic repulsion, steric stabilization and network formation [21,22,23]. These interventions are not universally beneficial: improvement of one property may be accompanied by aggregation, oxidation, loss of solubility, reduced gel or foam stability, or sensory defects. The appropriate strategy must therefore be selected according to the protein fraction, intended function and final food matrix.

Enzymatic hydrolysis and fermentation-assisted modification are also used to generate peptide-rich fractions. Egg-derived peptides and hydrolysates have demonstrated antioxidant, anti-inflammatory, antihypertensive, antimicrobial, immunomodulatory, intestinal barrier-related and mineral-binding potential [24,25,26,27,28]. Most evidence for these activities remains preclinical. Identification of a peptide sequence or activity in an in vitro assay does not demonstrate a physiological effect. Biological relevance depends on peptide release, gastrointestinal stability, bioaccessibility, intestinal transport, effective concentration and interactions with the food matrix [29,30,31,32,33]. Techno-functional properties such as foaming, emulsification and gelation should therefore be distinguished from biological or nutritional outcomes, even though both dimensions influence ingredient development.

Processing also determines the gastrointestinal behavior of egg proteins. Heating, non-thermal treatments, emulsification and gelation can modify aggregation, network density and accessibility to digestive enzymes [34,35,36,37]. These effects are relevant to foods developed for older adults and consumers with specific nutritional or textural requirements, for whom protein quality, digestibility, texture and acceptance must be considered together [38,39,40]. Human evidence remains limited, although intervention data for selected egg-protein hydrolysates indicate that physiological effects may be demonstrated when the preparation, dose and endpoints are adequately characterized [41]. Such findings cannot yet be generalized to the wider range of egg-derived peptides and modified protein ingredients.

Safety and consumer acceptance provide additional constraints. Egg contains clinically relevant allergens, and processing cannot be assumed to eliminate allergenic potential [42,43,44]. Depending on the protein fraction, epitope structure, matrix and treatment conditions, processing may reduce, increase, mask or leave immunoglobulin E (IgE)-binding capacity unchanged [45,46]. Reduced immunoreactivity in vitro is not equivalent to clinical tolerance, and reliable assessment may require the measurement of several egg allergens in processed foods [47]. Allergen labeling, bitterness, color changes, off-flavors, batch variability, regulatory requirements, process cost and scalability must therefore be considered together with technological performance [48,49,50,51].

Previous reviews have addressed individual topics such as egg white functionality, egg yolk emulsions, gelation, delivery systems, bioactive peptides, digestibility or allergenicity. The evidence remains divided among studies of protein structure, processing, peptide release, biological activity and food application. This separation makes it difficult to determine whether an improvement observed in a model system can be reproduced in a complete food and maintained during processing and storage. A combined assessment is needed to connect protein source and modification strategy with structural change, technological performance, digestion, safety and application-specific limitations.

This structured narrative review critically examines egg-derived proteins as functional food ingredients, with emphasis on enzymatic and fermentation-assisted modification, physical technologies, and chemical or biochemical strategies. It integrates protein source, modification mechanism, structural and peptide-level changes, digestibility, bioavailability, techno-functional performance, biological relevance, allergenicity, sensory quality, scalability and food application. In this review, “egg proteins” refers primarily to proteins derived from chicken (*Gallus gallus domesticus*) egg white and yolk. Evidence from other avian species, particularly duck eggs, is included selectively, and the species is identified when the reported evidence was not obtained from chicken eggs. By connecting molecular and structural findings with application-specific evidence, the review identifies both the potential of modified egg proteins and the requirements that remain before their use as reproducible, safe and acceptable functional food ingredients.

## 2. Materials and Methods

This article was designed as a structured narrative review focused on egg-derived proteins as functional food ingredients. The literature search was conducted during manuscript preparation and was last updated on 22 September 2026. All references assigned to the 2026 publication year had been formally published online and provided with final bibliographic information, including a volume and page range or article number, by the date of the final search. No unpublished manuscripts or references listed solely as “in press” were included. Relevant publications were identified using the bibliographic databases Scopus, Web of Science and PubMed, together with searches of the ScienceDirect, Wiley Online Library, SpringerLink and the Multidisciplinary Digital Publishing Institute (MDPI) platform.

The search focused primarily on peer-reviewed publications published between 2020 and 2026 to reflect recent developments in egg-protein modification, bioactive peptide research, gastrointestinal digestion, bioaccessibility, bioavailability, techno-functional properties, allergenicity and functional food applications. Earlier publications were included selectively when they provided essential methodological, regulatory, industrial or product-specific information, particularly for subjects such as powdered egg storage, packaging and product stability.

The search strategy combined terms related to protein source, modification technology, biological activity, digestion, safety and food application. The main terms included “egg-derived proteins”, “egg white proteins”, “egg yolk proteins”, “ovalbumin”, “ovotransferrin”, “ovomucoid”, “lysozyme”, “ovomucin”, “phosvitin”, “lipovitellins”, “low-density lipoproteins”, “high-density lipoproteins”, “enzymatic hydrolysis”, “fermentation”, “protein modification”, “pH-shifting”, “ultrasound”, “high-pressure processing”, “pulsed electric fields”, “cold plasma”, “microwave treatment”, “irradiation”, “spray-drying”, “glycation”, “Maillard conjugation”, “phosphorylation”, “succinylation”, “protein–polyphenol interactions”, “bioactive peptides”, “digestibility”, “bioavailability”, “bioaccessibility”, “intestinal absorption”, “emulsifying properties”, “foaming properties”, “gelling properties”, “delivery systems”, “functional foods”, “nutraceutical delivery”, “allergenicity”, “sensory quality”, “powdered eggs”, “packaging”, “storage stability” and “industrial scalability”. Species-related terms, including “chicken egg”, “hen egg”, “duck egg” and “avian egg”, were used where relevant. Additional terms included “eggshell membrane”, “egg-processing by-products” and “egg-processing co-products”.

The search terms were combined into three main concept groups: protein source, modification strategy, and functional or biological outcome. An example of the Boolean search structure was: (“egg protein” OR “egg white protein” OR “egg yolk protein” OR ovalbumin OR ovotransferrin OR “egg-derived peptide” OR “chicken egg” OR “hen egg” OR “duck egg” OR “eggshell membrane” OR “egg-processing by-product” OR “egg-processing co-product”) AND (hydrolysis OR fermentation OR glycation OR phosphorylation OR succinylation OR ultrasound OR microwave OR “high-pressure processing” OR “cold plasma” OR “thermal treatment”) AND (solubility OR foaming OR emulsification OR gelation OR digestibility OR bioavailability OR bioactivity OR allergenicity). The terminology and syntax were adapted to the requirements of each database or publishing platform. The reference lists of relevant publications were also examined to identify additional studies within the scope of the review.

Chicken egg white and yolk proteins constituted the primary scope of the review. Studies involving proteins or peptides derived from other avian species, particularly duck eggs, were included selectively when they provided directly relevant comparative or complementary evidence on modification mechanisms, structural behavior, biological activity or technological functionality. Evidence concerning eggshell membrane and other egg-processing fractions was included only when the investigated material contained protein or peptide components relevant to functional food, nutraceutical or food-delivery applications. The avian species and specific egg-derived fraction are explicitly identified throughout the manuscript whenever the evidence was not obtained from chicken egg white or yolk.

Articles were included when they directly addressed egg white or yolk proteins, defined egg-protein fractions, egg-derived peptide systems or the additional protein-containing materials specified above. Eligible studies provided relevant information on structural modification, peptide release, gastrointestinal digestion, bioaccessibility, bioavailability, techno-functional behavior, biological activity, allergenicity, safety, sensory limitations, storage stability or food applications. Review articles were used when they provided comprehensive syntheses of specific subjects, including egg white functionality, egg yolk emulsions, gelation, delivery systems, bioactive peptides, digestibility, allergenicity and food-grade modification technologies. Original research articles were prioritized when they reported recent experimental evidence concerning modification mechanisms, structural changes, functional performance, digestion behavior or food applications.

Studies focused exclusively on general egg nutrition, poultry production, shell quality, freshness assessment, omega-3/omega-6 enrichment, microbiological safety or meat quality were excluded unless they provided evidence directly relevant to the functionality, stability or food application of egg-derived proteins. Studies on egg packaging or powdered egg storage were included only when they contributed to understanding the stability, composition or technological relevance of egg-derived ingredients used in food systems.

The selected literature was organized according to five interconnected dimensions: protein source, modification strategy, structural or peptide-level change, functional or biological outcome, and food application. This framework connected egg white and yolk proteins with enzymatic, fermentation-assisted, physical, chemical and biochemical modification strategies and supported evaluation of their effects on digestibility, bioavailability, emulsification, foaming, gelation, solubility, biological activity, allergenicity and functional food development. Particular attention was given to limitations affecting translation into commercial products, including incomplete peptide standardization, limited human validation, sensory quality, clean-label acceptability, regulatory considerations, reproducibility, storage stability and industrial scalability.

Because this work was designed as a structured narrative synthesis rather than a systematic review or meta-analysis, the literature was not selected through a formal Preferred Reporting Items for Systematic Reviews and Meta-Analyses (PRISMA)-based protocol. Studies were evaluated according to their relevance to egg-derived proteins, publication date, methodological clarity, contribution to understanding structure–function relationships and applicability to functional food development. This approach supported an integrated and critical synthesis of current knowledge rather than a quantitative assessment of effect sizes.

## 3. Composition, Structure, and Functional Potential of Egg-Derived Proteins

Egg-derived proteins form a heterogeneous biological system distributed mainly between egg white and egg yolk. Their relevance results from the combined behavior of proteins, glycoproteins, phosphoproteins and lipid–protein assemblies that differ in molecular organization, thermal stability, surface activity and susceptibility to enzymatic hydrolysis. These characteristics support techno-functional properties such as foaming, gelation, emulsification, water binding and interfacial stabilization. They also determine biological or nutritional functions, including mineral binding, antimicrobial activity and the release of bioactive peptides [1].

Table 1 summarizes the principal egg-derived proteins and fractions relevant to functional food design, including their structural features, techno-functional properties, biological or nutritional relevance and practical limitations. Figure 1 illustrates the relationships between the major egg white and egg yolk components and their respective technological and biological roles.

### 3.1. Egg White Proteins: Composition and Functional Relevance

Egg white is an aqueous system containing proteins with different molecular sizes, isoelectric points, glycosylation patterns, thermal sensitivities and biological functions. Its principal components include ovalbumin, ovotransferrin, ovomucoid, ovomucin, lysozyme and avidin, together with minor proteins and protease inhibitors [1,52]. Their relative abundance and structural diversity account for the gelation, foaming, emulsification and viscosity of egg white, while individual proteins also contribute antimicrobial activity, allergenic potential and bioactive peptide precursor functions, as summarized in Table 1 and Figure 1.

Ovalbumin, the most abundant egg white protein, is a major contributor to gelation, foaming and emulsion stabilization. Its technological behavior is associated with its capacity to unfold, expose reactive groups and establish intermolecular interactions during heating or other treatments [6,53,54]. However, industrial applications generally use whole egg white or mixed protein fractions rather than purified ovalbumin because purification is costly and interactions among egg white proteins affect their collective behavior [52,53]. Results obtained using isolated ovalbumin must therefore be interpreted in relation to the composition of the ingredient under investigation.

Ovotransferrin combines technological and biological functions. Its metal-binding capacity contributes to antimicrobial activity, while peptides released from this protein have been associated with antioxidant, antimicrobial and antihypertensive effects [55]. Processing-induced changes in ovotransferrin may also influence gel formation and interfacial behavior. Ovomucoid is distinguished by its stability, resistance to digestion and relevance to egg allergy [42,43,44,45,46,47]. Lysozyme contributes antimicrobial activity, whereas ovomucin supports the viscosity of thick egg white and the stability of foams through its large glycoprotein structure [1,2,52,55]. Avidin has a more limited technological role but remains nutritionally relevant because of its strong affinity for biotin [1,52].

The collective capacity of egg white proteins to form gels, foams, films and interfacial layers supports their use as structuring agents, food hydrocolloids and delivery materials [6,7,8]. Limitations such as pH sensitivity, heat-induced aggregation and loss of solubility provide the technological basis for the modification strategies discussed in Section 4, Section 5 and Section 6.

### 3.2. Egg Yolk Proteins and Lipoprotein Fractions

Egg yolk is more structurally complex than egg white because many of its proteins occur within lipid-rich supramolecular assemblies. Its main protein-containing components include low-density lipoproteins, high-density lipoproteins, lipovitellins, livetins and phosvitin [1,4,5,56]. Yolk therefore functions both as a protein source and as a naturally organized lipid–protein emulsion. Its technological behavior depends on protein composition, phospholipids, lipid–protein interactions and the organization of plasma and granule fractions.

The plasma fraction is rich in low-density lipoproteins and livetins, whereas granules contain high-density lipoproteins, lipovitellins and phosvitin associated through phosphocalcic bridges [4,56]. Low-density lipoproteins make a major contribution to emulsification through adsorption and film formation at oil–water interfaces [3,5,56]. Yolk granules generally have lower solubility under some processing conditions but remain relevant as protein-rich ingredients with emulsifying, heat-resistant and structuring properties [4,56].

The high phosphorylation level of phosvitin accounts for its strong affinity for metal ions and its potential antioxidant, mineral-binding and nutrient-delivery functions [4,57]. Phosvitin-derived phosphopeptides may extend these applications, although food-grade recovery is constrained by extraction efficiency, cost and solubility. More broadly, the interfacial behavior of yolk proteins and lipoproteins is influenced by pH, ionic strength and processing conditions [3,5]. Their natural affinity for lipid-containing systems supports their application in emulsified foods and in delivery systems for lipophilic compounds [5,9].

### 3.3. Structure–Function Relationships

The functional properties of egg-derived proteins are governed by their structural state rather than solely by their concentration in a food matrix. Secondary-structure organization, tertiary conformation, molecular flexibility, surface charge, exposed hydrophobic regions, sulfhydryl availability, particle size and aggregation state determine protein solubility, interfacial adsorption and the capacity to form cohesive foam, emulsion or gel networks. Moderate protein unfolding may expose previously buried hydrophobic and reactive groups and increase molecular flexibility, facilitating adsorption at air–water or oil–water interfaces. In contrast, excessive unfolding followed by uncontrolled aggregation can decrease solubility, restrict molecular diffusion and produce heterogeneous interfacial films or gel networks [2,3,6,7].

Modification treatments provide several documented examples of these relationships. During pH-shifting, exposure to strongly acidic or alkaline conditions promotes unfolding, followed by partial refolding after neutralization. The resulting changes in particle size, surface hydrophobicity, charge distribution and molecular flexibility can facilitate adsorption at the oil–water interface and improve the emulsifying capacity and stability of egg white proteins [15]. Controlled enzymatic hydrolysis reduces molecular size and may increase solubility and diffusion toward the air–water interface. Moderate hydrolysis has consequently been associated with improved foam formation, whereas extensive hydrolysis may produce peptides that are too small to form cohesive and mechanically resistant interfacial films [16]. Ultrasound-generated cavitation can disrupt aggregates and induce partial protein unfolding, thereby increasing the accessibility of surface-active regions and improving the foaming properties of pasteurized liquid egg white. However, treatment intensity must be controlled because excessive structural disruption or reaggregation may reduce the expected functional benefit [17].

Changes in secondary and tertiary structure also affect thermal and interfacial behavior. In succinylated ovalbumin, the α-helix content decreased from 28.5% to 21.3%, whereas the β-sheet content increased from 28.1% to 43.0%. These changes were accompanied by tertiary-structure unfolding, increased exposure of sulfhydryl groups, greater electronegativity and improved solubility and interfacial behavior [23]. The increase in β-sheet organization was associated with stronger intermolecular hydrogen bonding and greater thermal stability. Phosphorylation introduces negatively charged phosphate groups, increasing electrostatic repulsion and protein hydration, which can improve solubility, foaming and emulsifying properties when the degree of modification is controlled [22]. Similarly, Maillard-type conjugation with polysaccharides can alter protein conformation while adding a hydrated steric layer, supporting interfacial stabilization and foam stability [21].

Gelation depends on a different balance between unfolding and association. Partial unfolding exposes hydrophobic regions and sulfhydryl groups that participate in hydrophobic interactions, hydrogen bonding and sulfhydryl–disulfide exchange during heating. These interactions promote the formation of a continuous three-dimensional network. Insufficient unfolding limits intermolecular association, whereas excessive aggregation before network formation may produce coarse, poorly hydrated and mechanically non-uniform gels [6]. Therefore, structural changes that improve emulsification or foaming do not necessarily improve gelation to the same extent.

Egg white and egg yolk proteins also differ in their structure–function relationships. Egg white proteins are more directly associated with foaming and heat-induced gelation, whereas yolk lipoproteins are strongly involved in adsorption and stabilization at oil–water interfaces [2,3,4,5,6]. Nevertheless, the functional response remains dependent on the surrounding matrix. Lipids, polysaccharides, salts and phenolic compounds can modify protein unfolding, aggregation and interfacial behavior [13,14]. Consequently, structural modification should be optimized according to the targeted function and subsequently validated in the intended food matrix rather than assessed only in simplified protein dispersions.

### 3.4. Relevance for Functional Food Design

Egg white and egg yolk provide complementary technological and biological functions in food design. Egg white proteins are principally suited to foaming, gelation, film formation and protein-based delivery systems, whereas yolk proteins and lipoproteins contribute mainly to emulsification and lipid structuring. From a biological or nutritional perspective, both fractions may serve as precursors of bioactive peptides, while phosvitin and its derived phosphopeptides provide mineral-binding potential [24,25,26,27,28,55,57]. This compositional foundation supports the evaluation in the following sections of how enzymatic, fermentation-assisted, physical, chemical and biochemical treatments can direct egg-derived proteins toward specific technological or biological applications.

## 4. Enzymatic and Fermentation-Assisted Modification of Egg Proteins

Enzymatic and fermentation-assisted treatments can modify egg-derived proteins through peptide-bond cleavage, cross-link formation, phospholipid transformation or microbial proteolysis. These processes affect molecular-weight distribution, solubility, interfacial behavior, gel formation and peptide release. Table 2 summarizes the principal strategies, while Figure 2 presents their mechanisms, resulting protein or peptide fractions and main technological or biological outcomes.

### 4.1. Enzymatic Hydrolysis as a Tool for Egg Protein Functionalization

Proteolytic hydrolysis converts egg proteins into polypeptides and peptides with properties determined by enzyme specificity, degree of hydrolysis, substrate concentration, pH, temperature, reaction time and substrate pretreatment. The studies considered in this review used defined preparations, including neutral protease [16] and Alcalase [58], while pepsin, trypsin, papain and other food-grade proteases have been reported for different egg-protein substrates [24,25,26,27,28]. Limited hydrolysis may increase solubility, molecular flexibility and interfacial adsorption or release peptides with antioxidant, anti-inflammatory, antimicrobial or ACE-inhibitory potential. Extensive hydrolysis may instead weaken interfacial films and gel networks and increase bitterness through the release of hydrophobic sequences [16,24,25,26,27,28,49,58].

The required degree of hydrolysis depends on the intended function. Neutral-protease treatment improved the foaming capacity of egg white protein under controlled conditions [16], whereas Alcalase hydrolysis generated peptide fractions with reproducible antioxidant activity and repeated sequences across production batches [58]. These findings represent different outcomes of the same general modification strategy rather than separate categories of enzymatic hydrolysis.

### 4.2. Egg White Protein Hydrolysates and Bioactive Peptide Release

Ovalbumin, ovotransferrin, lysozyme, ovomucin and ovomucoid can act as precursors of biologically active peptides. Ovotransferrin-derived hydrolysates have received particular attention for their ACE-inhibitory, antihypertensive, antioxidant and antimicrobial potential [24,55,59]. However, the technological and biological properties of a hydrolysate depend on peptide size, sequence and degree of hydrolysis. Conditions selected to increase peptide bioactivity may not preserve the molecular dimensions required for foam, emulsion or gel stabilization.

Peptide profiles can also vary with enzyme source, enzyme-to-substrate ratio, pretreatment, reaction conditions and downstream fractionation. The batch assessment reported in [58] illustrates the importance of monitoring degree of hydrolysis, antioxidant activity and peptide-sequence reproducibility when hydrolysates are proposed as standardized functional ingredients.

The gastrointestinal stability of egg-derived peptides is a major determinant of their potential in vivo activity. Following ingestion, peptide-rich hydrolysates are exposed to gastric acidity, pepsin, pancreatic proteases and intestinal brush-border peptidases. These conditions may completely degrade an active sequence, convert it into shorter fragments with reduced or different activity, or release new bioactive sequences from larger precursor peptides. Resistance to digestion depends on peptide length, amino acid sequence, conformation, terminal residues and interactions with other components of the food matrix [29,30,31,32,33]. The matrix may protect some peptides from rapid proteolysis, but it may also restrict their release and intestinal accessibility. Moreover, peptide persistence during simulated gastrointestinal digestion should not be interpreted as evidence of in vivo bioavailability. To produce a physiological effect, intact peptides or active fragments must reach the intestinal surface, resist brush-border degradation, cross the epithelium and retain activity after absorption and further metabolism. Egg-specific evidence completing this sequence remains limited, as most available studies rely on in vitro digestion, enzyme-inhibition assays, cell models or animal experiments. Therefore, gastrointestinal stability, intestinal transport and systemic exposure should be evaluated together before biological efficacy is attributed to an egg-derived peptide preparation. These aspects are discussed further in Section 7.

### 4.3. Enzymatic Modification of Egg Yolk Proteins and Lipoproteins

The response of egg yolk to enzymatic treatment depends on both protein hydrolysis and changes in lipid–protein organization. Papain, lipase and a flavor protease preparation have been reported to alter protein conformation, sulfhydryl exposure, gel texture and the binding or release of volatile compounds [60,61]. These effects distinguish yolk modification from the predominantly peptide-oriented hydrolysis of egg white.

Phospholipase A1 or A2 treatment modifies the phospholipid environment of yolk lipoproteins rather than primarily cleaving protein peptide bonds. The resulting reorganization of lipoprotein structures can increase protein solubility, thermal stability and emulsifying performance [62]. Phosvitin hydrolysis provides a further route by releasing phosphorylated peptides with mineral-binding and antioxidant potential [57,64]. Its practical use remains affected by recovery yield, purification requirements, solubility and production cost.

### 4.4. Fermentation-Assisted Modification

Fermentation combines microbial proteolysis with acidification and metabolite formation. Lactic acid bacteria have been used to modify egg-derived ingredients through changes in protein structure, peptide release, particle size and digestibility [20]. In spray-dried egg yolk powder, fermentation increased solubility, water dispersibility, dispersion stability and simulated gastrointestinal digestibility [63].

Egg-specific evidence nevertheless remains limited compared with the literature on enzymatic hydrolysis and physical modification. Fermentation-assisted modification should therefore be regarded as an emerging approach whose reproducibility, strain dependence and applicability to different egg-derived fractions require further investigation.

The final properties depend on the microbial strain, substrate composition, fermentation time, pH evolution and subsequent drying. Acidification and microbial metabolites may also modify aroma and taste. Fermentation studies should therefore report both compositional changes and the resulting technological and sensory properties, particularly when peptide generation is proposed as a functional outcome.

### 4.5. Enzymatic Cross-Linking

Transglutaminase-mediated cross-linking differs from hydrolysis because it forms covalent bonds between accessible glutamine and lysine residues. The compact tertiary structure of native globular egg white proteins may limit enzyme access to these residues, while partial unfolding induced by heat or reducing pretreatment can increase their availability [65,66]. Controlled cross-linking may strengthen protein networks and improve water retention, whereas an excessive reaction may produce rigid structures with reduced solubility.

The degree of cross-linking is therefore the principal technological variable in transglutaminase-treated egg proteins. Insufficient cross-linking may produce little change in network strength, while extensive cross-link formation may alter protein accessibility to digestive enzymes. However, direct evidence demonstrating that transglutaminase-mediated cross-linking reduces the digestibility of egg-derived proteins remains limited. Digestibility should therefore be measured rather than inferred from network rigidity or reduced solubility. Treatment efficiency should be evaluated using cross-link density, rheological behavior, water-holding capacity, syneresis, fracture properties and appropriate digestion assays. Enzyme concentration, reaction time and structural pretreatment should also be reported because these variables determine the accessibility of glutamine and lysine residues and the resulting network architecture [65,66].

## 5. Physical Modification Technologies

Physical technologies can modify egg-protein conformation, aggregation, particle size and interfacial behavior without primarily cleaving peptide bonds. Depending on the treatment conditions, they may improve gelation, foaming, emulsification, solubility, microbial stability or powder reconstitution. Their effects are determined by treatment intensity, duration, temperature, moisture, pH, matrix composition and protein fraction. Insufficient treatment may produce limited structural change, whereas excessive processing can cause irreversible aggregation, oxidation, solubility loss or sensory defects. Table 3 summarizes the principal technologies, and Figure 3 presents their main structural pathways and functional consequences.

### 5.1. Thermal Processing

Thermal processing contributes to microbial safety, coagulation, gelation and food structuring. Heating unfolds globular egg proteins and promotes aggregation through hydrophobic interactions, hydrogen bonding and disulfide exchange. Moderate treatment may support network formation and digestive-enzyme accessibility, whereas excessive heating can generate compact aggregates with reduced solubility and limited accessibility during gastrointestinal digestion [34,35,67].

Prolonged boiling increased hardness and chewiness but reduced digestibility and antioxidant-peptide release, particularly in egg white, through the formation of a compact gel network [67]. This result illustrates an application-specific trade-off: firm networks may be desirable in structured foods, whereas softer matrices may be more appropriate for older adults, individuals with dysphagia or products designed for greater protein accessibility [36,37,38,39,40]. Thermal conditions should therefore be selected according to the required texture, product format and nutritional objective. Detailed relationships among structure, digestion and bioavailability are discussed in Section 7 and Section 8.

Schematic representation of heat treatment, ultrasound, high-pressure processing, pulsed electric fields, cold plasma, microwave treatment, irradiation and drying, together with their effects on protein unfolding, aggregation and supramolecular organization. The resulting changes may affect solubility, foaming, emulsification, gelation, digestibility and microbial stability. Principal constraints include oxidation, irreversible aggregation, non-uniform treatment, sensory changes and scale-up requirements.

### 5.2. High-Pressure Processing

High-pressure processing disrupts non-covalent interactions and can induce protein unfolding, aggregation and gel formation with a lower thermal load than conventional heating. Mild high hydrostatic pressure modified ovalbumin conformation, foaming capacity, emulsifying activity and allergenicity under selected conditions [19]. In an application-oriented study, pressure-induced gelation improved the texture, syneresis and microstructure of egg-white pudding [68], indicating potential for soft, protein-rich foods.

The resulting properties depend on pressure level, holding time, temperature and matrix composition. Excessive treatment may produce dense networks or undesirable sensory changes, and results obtained using purified ovalbumin cannot be assumed to apply directly to whole egg white, yolk or composite foods. Product assessment should therefore include texture, stability, sensory quality and economic feasibility. Digestion-related effects and allergenicity are considered in Section 8 and Section 10, respectively.

### 5.3. Ultrasound Treatment

Ultrasound treatment has received considerable attention as a physical method for modifying egg white proteins. Its effects are mainly attributed to acoustic cavitation, local shear forces, turbulence and microstreaming, which can disrupt weak aggregates, reduce particle size and promote partial unfolding. These structural changes may increase protein solubility, expose reactive groups and improve adsorption at air–water or oil–water interfaces.

Ultrasound modification has been reported to improve the foaming properties and storage stability of pasteurized liquid egg white [17]. Under optimized conditions, ultrasound promoted protein deaggregation, increased solubility, reduced particle size, enhanced free sulfhydryl exposure and surface hydrophobicity, and improved foaming capacity. This is relevant because pasteurization is often necessary for liquid egg safety, but it can compromise foaming performance. Ultrasound may therefore serve as a post-pasteurization or combined treatment to recover or improve functionality.

Ultrasound intensity and treatment duration must be carefully controlled. Mild to moderate treatment can promote protein unfolding, deaggregation and dispersion. In contrast, excessive sonication may induce oxidation and protein reaggregation, decreasing solubility, foaming capacity, foam stability and emulsifying performance. Industrial application also depends on the reactor design, energy distribution and treatment uniformity at larger processing volumes [17].

### 5.4. Pulsed Electric Fields and Cold Plasma

Pulsed electric fields may alter charge distribution, non-covalent interactions and protein conformation. However, recent research remains dominated by dairy and plant proteins, and direct evidence for egg-derived proteins is limited [69]. Its use for egg ingredients should therefore be regarded as emerging until processing conditions and food applications are supported by additional egg-specific studies.

Cold plasma has been evaluated more directly in egg white systems. Reactive species generated during treatment can modify protein conformation, solubility, foaming, emulsifying and gelling properties and may contribute to microbial control [18]. Excessive exposure may oxidize amino acid residues, affect flavor or reduce nutritional quality. Treatment uniformity, oxidation markers, sensory effects, allergenicity and scale-up therefore require application-specific evaluation.

### 5.5. Microwave, Irradiation and Drying-Related Modifications

Microwave processing produces rapid volumetric heating and associated structural transitions. Controlled microwave treatment improved the foaming properties of egg white powder [70], but uneven temperature distribution may cause localized overheating, aggregation and solubility loss.

Selected irradiation doses increased the hydrophobicity, foaming capacity and heat-set gelation of liquid egg white [71]. Higher doses may instead promote oxidation and uncontrolled aggregation, indicating that dose selection must balance interfacial or gel-forming performance against chemical and sensory changes.

Drying is directly relevant to egg white, whole-egg and peptide powders. Spray-drying conditions can affect protein aggregation, hydrophobic-group exposure, rehydration and gel formation [72], while subsequent ingredient stability also depends on packaging and storage conditions [11,12]. Inlet temperature and flow rate modified the physicochemical and gelling properties of egg white protein, with excessive thermal exposure impairing homogeneous network formation [72]. In salted duck egg white peptide powder, freeze-drying preserved solubility, foaming, emulsification and antioxidant activity more effectively than vacuum drying or spray-drying [73]. This duck-specific evidence illustrates the influence of drying intensity but should not be generalized directly to chicken egg ingredients.

### 5.6. Combined Physical Modification Strategies

Recent studies have investigated sequential or simultaneous physical treatments because different technologies may act through complementary mechanisms. One treatment may promote controlled unfolding or reduce particle size, while a second treatment may stabilize the modified structure or regulate aggregation. However, combined treatments should not be assumed to be synergistic unless they are compared with the corresponding individual treatments. Treatment order, temperature, energy input and duration determine whether the resulting changes are beneficial or lead to excessive denaturation and aggregation.

Preheating combined with high-intensity ultrasound altered the secondary structure, sulfhydryl exposure, surface hydrophobicity and viscosity of ovalbumin. Treatment at 200 W and 60 °C produced the highest foaming capacity and stability among the tested conditions, indicating that moderate preheating increased the susceptibility of ovalbumin to ultrasound-induced structural modification [74]. Ultrasound–microwave treatment has also been used to prepare egg white microgels. In this system, ultrasound limited the excessive aggregation caused by microwave heating and produced particles of approximately 233 nm with suitable surface and interfacial properties for stabilizing Pickering emulsions [75].

A sequential ultrasound–heating pretreatment applied to liquid egg white before drying also improved the properties of the resulting powder. Ultrasound followed by mild heating increased foaming stability, emulsifying activity and emulsifying stability and improved in vitro digestibility, although the magnitude of the effect depended on whether freeze-drying or spray-drying was subsequently used [76]. These findings indicate that combined physical treatments can control several structural transitions within the same process, but their effectiveness depends on the sequence and intensity of each treatment.

## 6. Chemical and Biochemical Modification Strategies

Chemical and biochemical strategies modify egg-protein charge, hydrophobicity, steric properties, covalent interactions and molecular flexibility. These changes can affect solubility, foaming, emulsification, gelation, thermal stability and delivery performance. Their use in foods depends on reaction control, reagent acceptability, formation of reaction products, sensory effects, ingredient identity and regulatory requirements. Table 4 summarizes the main approaches, and Figure 4 presents their molecular pathways and principal constraints.

Schematic representation of pH-shifting, Maillard-type glycation, phosphorylation, succinylation and protein–polyphenol interactions. These strategies modify charge distribution, hydrophobicity, steric stabilization and intermolecular interactions, thereby affecting solubility, foaming, emulsification, gelation, mineral binding and antioxidant properties. Principal constraints include browning, irreversible cross-linking, residual reagents, sensory changes and process-control requirements.

### 6.1. pH-Shifting and Controlled Charge Modulation

During pH-shifting, proteins are exposed to acidic or alkaline conditions and subsequently returned to a neutral or application-relevant pH. The resulting changes in surface charge and non-covalent interactions can promote partial unfolding, expose hydrophobic or sulfhydryl groups and modify dispersion and interfacial behavior. Alkaline pH-shifting improved the emulsifying activity and emulsion stability of egg white proteins through changes in molecular structure, surface hydrophobicity and interfacial adsorption [15]. Preheating followed by pH-shifting also reduced particle size and improved the solubility, interfacial elasticity and heat stability of egg white protein particles [77].

Industrial application requires control of pH severity, neutralization, salt formation, water use and process reproducibility. Extreme conditions may produce irreversible aggregation, nutritional changes or undesirable sensory effects. The technological benefit must therefore be confirmed under processing conditions relevant to the intended food.

### 6.2. Glycation and Maillard-Type Protein–Polysaccharide Conjugation

Among chemical and biochemical strategies, Maillard-type glycation has received considerable attention because it can improve protein functionality using food-compatible carbohydrates. In this approach, reducing sugars or polysaccharides react with free amino groups of proteins, mainly those of lysine residues, leading to the formation of covalent protein–carbohydrate conjugates. In egg protein systems, glycation can increase steric stabilization, modify surface hydrophobicity, alter thermal aggregation behavior and improve foaming, emulsifying or gelation properties.

Ovalbumin–pullulan conjugates produced through the Maillard reaction showed covalent attachment and improved foaming stability, illustrating the capacity of polysaccharide conjugation to modify the interfacial behavior of egg proteins [21]. Ultrasound-assisted Maillard glycation of ovalbumin with xylose further demonstrated that combined physical and chemical treatment can increase the degree of glycation and improve foaming and emulsifying properties by promoting unfolding and changes in surface characteristics [78]. These results suggest that glycation is not merely a chemical attachment reaction, but also a route for regulating molecular flexibility, surface activity and aggregation during food processing.

Glycation of egg white protein with maltodextrin in the dry state has also been reported to improve gel properties, including gel hardness, water-holding capacity, rheological behavior and microstructure [79]. More recently, ovalbumin–citrus pectin conjugates prepared by the Maillard reaction showed structural changes and improved foaming properties, supporting the potential of pectin-based conjugation for aerated food systems [80]. Egg white protein–dextran conjugates have also been investigated as emulsifying systems, with results showing that polysaccharide type, protein-to-polysaccharide ratio and reaction conditions are decisive for their final functionality [81].

Optimization should begin with the intended functional property. Neutral polysaccharides such as pullulan, dextran and maltodextrin can provide hydrated steric layers that limit protein aggregation and support foam or emulsion stability [21,79,81]. Anionic polysaccharides such as pectin additionally modify surface charge and interfacial-film viscosity, making them suitable for foam stabilization [80]. Small reducing sugars generally react more rapidly because their carbonyl groups are more accessible, but they provide less steric protection and may promote faster browning. Polysaccharides with higher molecular weights form thicker interfacial layers but may react more slowly because of steric limitations. Comparative work with glucose, fructose, xylose, maltodextrin and oligosaccharides confirms that different carbohydrate types result in different degrees of glycation and emulsion stability [87].

The protein-to-carbohydrate ratio should be selected according to the number of accessible protein amino groups, available reducing ends and targeted degree of grafting rather than using a single ratio for all systems. For example, published egg-protein studies have used an ovalbumin-to-citrus-pectin ratio of 1:2 and an egg white-protein-to-sugar ratio of 1:1, but these values represent formulation-specific starting points rather than universal optima [80,87]. Increasing the carbohydrate concentration may initially increase grafting, whereas an excessive amount can leave unreacted carbohydrate, alter viscosity or promote aggregation and solubility loss. Optimization should therefore involve monitoring the loss of free amino groups, degree of grafting, browning index, solubility and the targeted foaming, emulsifying or gelling property. Reaction temperature, duration, pH and water activity must be controlled together to favor early-stage conjugation while limiting advanced Maillard products and nutritional losses.

Despite these advantages, Maillard glycation has important limitations. Reaction temperature, water activity, duration, carbohydrate type and protein structure determine whether the process produces functional early-stage conjugates or progresses toward excessive browning and the formation of potentially undesirable advanced Maillard products, including advanced glycation end products (AGEs). Prolonged or poorly controlled reactions may reduce nutritional quality by blocking lysine residues and may generate undesirable color, flavor and reaction by-products. Process evaluation should therefore include the degree of glycation, loss of free amino groups, available lysine, browning index, formation of advanced reaction products and targeted functional property. Glycation conditions should be optimized for nutritional retention, sensory quality and food safety rather than for foaming, emulsification or gelation alone.

### 6.3. Phosphorylation

Phosphorylation introduces negatively charged phosphate groups that can increase hydration, electrostatic repulsion and mineral-binding capacity. Its relevance to egg proteins is also illustrated by phosvitin, a naturally phosphorylated yolk protein with strong mineral-binding properties [57]. Microwave-assisted phosphorylation modified the solubility, foaming, emulsifying, water- and oil-holding properties, viscosity and particle-size distribution of freeze-dried egg white powder [22]. The outcome depended on phosphate concentration and microwave power, and excessive treatment could produce aggregation or heterogeneous modification.

Phosphorylation following pulsed-electric-field pretreatment also altered ovalbumin conformation and reduced immunoglobulin G and immunoglobulin E binding in immunological assays [82]. This finding indicates modified immunoreactivity but does not demonstrate clinical hypoallergenicity. The allergenicity implications of processing-induced structural changes are examined in Section 10.

Translation of phosphorylation to commercial food production also requires identification of the phosphate reagent, degree of substitution and residual unreacted phosphate, together with any salts or reaction by-products generated during treatment and neutralization. Potential effects on amino acid availability, digestibility, total phosphorus content and sensory quality should be assessed. The regulatory status and appropriate ingredient designation of the resulting preparation depend on the reagent, processing conditions, intended use and jurisdiction and cannot be inferred from the natural occurrence of phosphorylated proteins such as phosvitin.

### 6.4. Acylation and Succinylation

Succinylation introduces negatively charged groups that increase electrostatic repulsion and molecular flexibility. Succinylated ovalbumin showed structural unfolding, improved solubility, reduced interfacial tension and improved foaming and emulsifying properties [23]. Succinylation of egg white protein hydrogels also modified water-holding capacity, hardness, elasticity and mineral-binding capacity [83], supporting their potential use in soft or mineral-enriched food matrices.

However, acylation and succinylation have clear limitations for food applications. Assessment should include the identity and food-grade status of the acylating reagent, degree of substitution, residual unreacted reagent, hydrolysis or reaction by-products and the method used to terminate and purify the reaction. Because modification of protein amino groups may affect amino acid availability, digestibility and ingredient identity, nutritional consequences should also be evaluated. Excessive succinylation may produce extensively modified proteins with undesirable sensory or functional characteristics. Regulatory classification and labeling must be determined for the specific preparation and intended market; therefore, laboratory-scale improvements cannot be assumed to translate directly into clean-label or conventional food applications.

### 6.5. Protein–Polyphenol Interactions and Covalent Conjugation

Polyphenols interact with egg proteins through hydrogen bonding, hydrophobic interactions and, following oxidation, covalent cross-linking. These interactions can alter conformation, aggregation, interfacial behavior and antioxidant properties. Conventional and ultrasound-assisted free-radical conjugation has been used to identify binding sites and characterize covalent modification patterns in egg white protein–polyphenol systems [85].

Egg-protein matrices containing orange and lemon peel polyphenols demonstrated antioxidant activity and lower immunoglobulin E binding than untreated proteins in enzyme-linked immunosorbent assays using sera from egg-allergic patients [86]. These findings indicate altered immunoreactivity under the tested conditions but do not establish clinical tolerance. Polyphenol composition, oxidation state, protein type, pH, processing and digestion may modify the response; the safety interpretation is therefore considered in Section 10.

Covalent protein–polyphenol conjugation also presents process-control and regulatory challenges. Methods based on phenolic oxidation or free-radical initiation should report the oxidizing or initiating system, conjugation yield, cross-linking degree, residual reagents, unbound polyphenols and oxidation-derived reaction products. Extensive conjugation may alter amino acid availability, digestibility, protein solubility, color, flavor or astringency. The composition and oxidation state of the polyphenol preparation must also be standardized because extracts from plant by-products may vary among sources and batches. Regulatory acceptability and ingredient labeling should therefore be established for the specific conjugate, production method and intended food application.

### 6.6. Comparative Assessment of Egg-Protein Modification Strategies

Egg-protein modification strategies differ in their intended functions, process requirements and translation constraints. Proteolytic hydrolysis is suited to peptide release and adjustment of solubility or interfacial behavior, although extensive hydrolysis may increase bitterness and reduce foam or gel stability. Phospholipase treatment primarily targets yolk emulsification and thermal stability, fermentation combines microbial proteolysis with compositional and sensory changes, and transglutaminase-mediated cross-linking strengthens protein networks. Fermentation remains less extensively investigated in egg-specific systems, and cross-linking requires control of network density and digestive-enzyme accessibility.

Physical treatments modify protein conformation, particle size and aggregation without direct chemical derivatization. Their performance depends on treatment intensity, temperature, energy input and matrix composition, and industrial implementation may be restricted by equipment cost, process uniformity and energy requirements. Chemical and biochemical approaches provide more targeted control over charge, steric stabilization and covalent interactions, but their use in foods requires consideration of reagent acceptability, reaction by-products, ingredient identity, sensory effects and regulatory status.

No strategy can be expected to improve technological performance, digestibility, biological activity and sensory quality simultaneously. Selection should therefore be based on the protein fraction, intended function, food matrix, downstream processing and evidence required for the proposed application. Comparisons remain difficult because studies frequently use different substrates, treatment conditions and analytical endpoints, often in purified proteins or model dispersions. Digestion and bioavailability are examined in Section 7 and Section 8, application-specific performance in Section 9, safety and allergenicity in Section 10, and industrial feasibility in Section 11.

## 7. Bioactive Peptides from Egg Proteins

Egg-derived proteins contain encrypted peptide sequences that may be released through enzymatic hydrolysis, microbial fermentation, gastrointestinal digestion or controlled processing. Parent proteins such as ovalbumin, ovotransferrin, lysozyme, ovomucin, phosvitin and lipovitellins can generate peptides with reported antioxidant, anti-inflammatory, antihypertensive, antimicrobial, immunomodulatory, gut-related or mineral-binding activities [24,25,26,27,28,55,57,64]. Protein-containing egg-processing fractions may also serve as substrates for peptide-rich ingredients, provided that their composition, safety and production reproducibility are adequately controlled [88].

The biological relevance of these peptides depends on more than their release from the parent protein. Enzyme specificity, degree of hydrolysis, peptide sequence, processing conditions and food-matrix interactions determine the composition and activity of the resulting preparations [16,24,25,26,27,28,58]. Most reported biological effects are supported by chemical assays, in vitro systems, cell models or animal studies, and only limited evidence is available from human interventions. Table 5 compares the principal peptide preparations and evidence levels reported in the literature. Figure 5 summarizes the progression from peptide production to biological relevance. Gastrointestinal stability, bioaccessibility and intestinal absorption are examined in detail in Section 8.

Egg-derived peptides may be released through processing, enzymatic hydrolysis, fermentation or gastrointestinal digestion. Evaluation of their biological relevance requires consideration of peptide composition, gastrointestinal stability, intestinal transport and systemic availability. Reported activities include angiotensin-converting enzyme (ACE) inhibition, and antioxidant, anti-inflammatory, antimicrobial and mineral-binding effects. Principal constraints include limited bioavailability, matrix interactions, sensory effects, batch variability and allergenicity.

### 7.1. Sources, Production and Identification of Egg-Derived Peptides

Egg white and egg yolk provide peptide precursors with different structural characteristics. Ovalbumin, ovotransferrin and lysozyme are frequently investigated because of their abundance and established peptide profiles [25,26,27,28,55]. Yolk proteins, including phosvitin, lipovitellins and other granule or plasma proteins, can generate antioxidant, antimicrobial and mineral-binding peptides [57,64]. Defatted yolk protein and protein-rich egg-processing fractions are also being investigated as substrates for peptide production [64,88].

Peptide preparation generally comprises protein recovery, controlled hydrolysis or fermentation, fractionation, sequence identification and biological screening. Ultrafiltration can separate fractions according to molecular size, whereas chromatographic and mass-spectrometric methods support peptide identification. In silico screening, molecular docking and network pharmacology may assist in selecting candidate sequences and proposing molecular targets [27,58], but predicted interactions require experimental confirmation.

Hydrolysates commonly contain complex peptide mixtures whose composition varies with the protein substrate, enzyme, hydrolysis conditions, pretreatment and downstream processing. Biological activity may result from several interacting sequences rather than from a single peptide. Consequently, batch reproducibility and sequence-level characterization are necessary when these preparations are proposed as functional ingredients [58]. Reporting should include the parent protein, enzyme preparation, degree of hydrolysis, molecular-weight distribution, principal peptide sequences and relevant processing conditions.

### 7.2. Antioxidant and Anti-Inflammatory Activities

Antioxidant activity is among the most frequently reported properties of egg-derived peptides. Proposed mechanisms include radical scavenging, metal chelation, inhibition of lipid oxidation and modulation of cellular oxidative stress pathways [25,26,58,64]. Ovalbumin, ovotransferrin, lysozyme and phosvitin can generate antioxidant peptide fractions, although their activity depends on peptide composition and the assay used. Peptides obtained from defatted egg yolk protein have also shown antioxidant and antimicrobial potential, demonstrating that yolk fractions provide relevant substrates in addition to egg white [57,64].

Anti-inflammatory effects have mainly been investigated in cell-based systems. Egg white protein-derived peptides have been associated with changes in inflammatory markers, oxidative stress responses and cytokine-related pathways [26,92]. Peptidomic analysis of hen egg white hydrolysate identified candidate anti-inflammatory sequences, some of which reduced inflammatory markers in lipopolysaccharide-stimulated macrophages [92]. These findings support further investigation of defined peptide sequences, but cell responses do not establish systemic efficacy. Physiological interpretation requires evidence that the active sequences remain available after digestion and reach relevant tissues at effective concentrations.

### 7.3. Antihypertensive and Cardiometabolic Potential

The antihypertensive potential of egg-derived peptides has most commonly been associated with inhibition of angiotensin-converting enzyme (ACE). Egg white hydrolysates may also affect vascular oxidative stress, endothelial function and renin–angiotensin-system regulation [24,59,89,90]. These additional mechanisms are relevant because ACE inhibition measured in a biochemical assay does not fully represent vascular responses in vivo.

Egg white protein hydrolysates reduced blood pressure or improved vascular parameters in spontaneously hypertensive and deoxycorticosterone acetate (DOCA)-salt rat models [59,89,90]. The reported responses were associated with pathways involving endothelial function, oxidative stress and regulation of the renin–angiotensin system. Nevertheless, differences in hydrolysate composition, dose and experimental model restrict direct comparison and extrapolation to humans.

Human evidence remains limited. A randomized, double-blind, placebo-controlled trial reported improved endothelial function following long-term consumption of an egg-protein hydrolysate by older adults with overweight or obesity [41]. This study provides evidence for the specific preparation, dose, population and outcome investigated, but does not establish a general effect for egg-protein hydrolysates. Additional controlled trials are required to determine dose–response relationships, long-term safety, inter-individual variability and efficacy when the preparations are incorporated into foods. Until such evidence is available, antihypertensive and cardiometabolic effects should be described as potential activities rather than confirmed clinical benefits.

### 7.4. Antimicrobial, Immunomodulatory and Gut-Related Activities

The antimicrobial potential of egg-derived peptides is supported by the presence of naturally occurring defense proteins in eggs, particularly lysozyme and ovotransferrin [55]. Enzymatic hydrolysis may release peptide fragments with antimicrobial properties that differ from those of the intact proteins [27,55,64,91]. Peptides obtained from egg white hydrolysates have demonstrated antimicrobial and anti-inflammatory activity against the acne-associated bacterium *Cutibacterium acnes* and the opportunistic skin pathogens *Staphylococcus aureus* and methicillin-resistant *S. aureus* (MRSA). Membrane disruption has been proposed as one of the mechanisms involved [91]. These results support the biological relevance of egg-derived peptides as multifunctional ingredients. However, activity demonstrated under experimental conditions cannot be directly interpreted as preservative efficacy in food products. Peptide activity may be affected by pH, salt concentration, fat content, water activity, interactions with other matrix components and the composition of the microbial population.

Egg yolk proteins also represent potential sources of antimicrobial peptides. Peptides obtained from defatted yolk protein have shown antioxidant and antimicrobial properties, indicating that yolk-processing fractions may provide substrates for the production of bioactive peptide ingredients [64]. Their recovery may also contribute to the valorization of protein-rich egg-processing streams that might otherwise be treated as low-value residues [88]. Further evaluation in food matrices is required to establish effective concentrations, stability during processing and storage, sensory effects and antimicrobial performance under conditions relevant to food production.

Evidence concerning the immunomodulatory and gut-related effects of egg-derived peptides remains at an early stage. Egg white proteins and their derived peptides have been investigated for possible effects on intestinal barrier function, inflammatory responses, oxidative stress and gut microbial composition [28]. Nevertheless, the available results are heterogeneous and are derived mainly from experimental models. Their biological effects may depend on peptide sequence and concentration, gastrointestinal stability, microbiota composition, host physiology, processing history and interactions within the food matrix. Further studies using standardized digestion models, physiologically relevant experimental systems and, where appropriate, human interventions are required before specific gut-health or immunomodulatory effects can be established.

### 7.5. Structure–Activity Relationships and Strength of Evidence

Peptide activity is influenced by amino acid sequence, molecular weight, hydrophobicity, charge, terminal residues and resistance to enzymatic degradation. Low-molecular-weight peptides may have greater opportunities for intestinal transport, but molecular size alone does not predict absorption or biological activity. Hydrophobic and charged residues can affect interactions with enzyme active sites, cell membranes, minerals or other molecular targets. Phosvitin-derived phosphopeptides are of particular interest for mineral binding because their phosphate-rich sequences provide multiple potential coordination sites [57,64].

Comparison of the available evidence reveals several recurring limitations. Many studies rely on chemical assays, enzyme-inhibition tests or cell models; hydrolysates are not always characterized at sequence level; and the stability, absorption and effective dose of identified peptides are rarely evaluated together. Differences in substrate composition, enzymes, hydrolysis conditions and analytical methods further restrict comparisons among studies. Sensory properties, especially bitterness, as well as ingredient interactions and batch reproducibility, are also insufficiently reported.

Egg-derived peptides should therefore be regarded as candidate bioactive ingredients rather than as preparations with established physiological efficacy. Progress toward food applications requires a connected evidence pathway encompassing controlled production, compositional characterization, digestion and transport studies, safety and allergenicity assessment, sensory evaluation and validation in realistic products. Section 8 examines the digestion, bioaccessibility and bioavailability components of this pathway, whereas Section 9 and Section 10 address application performance and safety considerations, respectively.

## 8. Digestibility, Bioavailability, and Food Matrix Interactions

Digestibility, bioaccessibility and bioavailability represent distinct stages in the nutritional evaluation of egg-derived proteins and peptides. Digestibility describes the extent of gastrointestinal protein hydrolysis. Bioaccessibility refers to the fraction of nutrients or peptides released from the food matrix and made available for intestinal uptake. Bioavailability additionally requires absorption, distribution to the relevant site and retention of activity in a physiologically relevant form [29,30,31,32,33]. Consequently, neither amino acid composition nor biological activity measured in vitro is sufficient to establish the physiological relevance of an egg-derived ingredient.

Processing history, aggregation state, food-matrix structure, storage and interactions with lipids, carbohydrates, minerals or phenolic compounds can affect protein hydrolysis and peptide release [29,30,31,32,33]. These factors may influence technological performance and nutritional accessibility in different directions. Table 6 summarizes the principal determinants of digestion and bioavailability. Peptide activities and their associated evidence levels are examined in Section 7, whereas this section focuses on gastrointestinal fate, transport and matrix-related effects.

### 8.1. Effect of Processing on Protein Digestibility

Processing can increase or reduce egg-protein digestibility according to the structural changes produced. Mild denaturation may expose cleavage sites to gastrointestinal enzymes, whereas extensive heating, oxidation or aggregation may generate compact networks that restrict enzyme penetration and hydrolysis [34,35]. The direction of the effect therefore depends on the treatment intensity, protein composition and matrix organization rather than solely on whether the process is thermal or non-thermal.

Boiling time affected gel formation, digestibility and antioxidant-peptide release from egg white and yolk [67]. Prolonged boiling increased network density and textural firmness but reduced digestibility and peptide release, particularly in egg white. This finding demonstrates that greater structural strength does not necessarily correspond to greater nutritional accessibility.

The physiological model used for assessment is also important. Cooking preparation and heat-induced gelation affected egg-protein digestion in models representing adult and older-adult gastrointestinal conditions [36,37]. Age-related changes in gastric acidity, enzyme activity and gastrointestinal transit may alter protein breakdown and peptide release. Ingredients intended for older consumers or individuals with modified digestive function should therefore be evaluated using physiologically relevant digestion conditions.

### 8.2. Peptide Release and Intestinal Transport

Peptides released during processing or gastrointestinal digestion may act locally within the gastrointestinal tract or, if absorbed, at peripheral sites. Their fate depends on the amino acid sequence, molecular size, hydrophobicity, charge, resistance to gastrointestinal and brush-border peptidases and interactions with intestinal transport pathways [29,33].

Egg-specific evidence for intact peptide absorption remains limited. Peptides derived from an egg white hydrolysate were detected in plasma following oral administration in an animal model, and clathrin-dependent endocytosis was proposed as an intestinal transport pathway [94]. This study indicates that selected peptides may cross the intestinal barrier in intact form, but it does not establish comparable absorption or biological efficacy in humans.

Cell models provide mechanistic information about intestinal responses but cannot reproduce the complete physiological context. Peptides released during digestion of heat-treated ovalbumin, ovomucoid and lysozyme produced different responses in Caco-2 cells according to the parent protein and processing history [95,96]. Some digestion-derived peptides were associated with inflammatory markers, demonstrating that peptide release should not automatically be interpreted as beneficial. Confirmation of biological relevance requires characterization of the post-digestion peptide profile, transport assessment and evidence obtained using physiologically appropriate models.

### 8.3. Role of the Food Matrix

Egg-derived proteins are generally consumed as components of gels, emulsions, bakery products, sauces, beverages, powders or composite ingredients. Matrix structure can modify enzyme accessibility, water mobility, molecular diffusion and peptide release [30,31,32]. Consequently, digestion results obtained from purified protein dispersions cannot be directly extrapolated to complete foods.

In gelled systems, network density and porosity regulate the penetration of digestive enzymes. In emulsions, interfacial organization and lipid–protein interactions can modify digestion kinetics. In bakery and confectionery products, starch, sugar and lipid interactions may influence protein accessibility. Egg yolk and whole-egg matrices are particularly complex because proteins are associated with lipids, phospholipids and lipoprotein assemblies [5,9,13]. Evaluation of these systems should therefore consider both protein digestion and changes occurring in the surrounding matrix.

Matrix effects also influence the interpretation of peptide bioactivity. A peptide that remains active in a purified solution may bind to other food components, undergo further hydrolysis or become unavailable for intestinal transport after incorporation into a product. Application-oriented studies should consequently combine food-matrix characterization with digestion and post-digestion peptide analysis.

### 8.4. Storage, Packaging and Powdered Egg Matrices

Drying, packaging and storage can modify the composition and subsequent performance of powdered egg ingredients. Changes in moisture, protein aggregation, lipid oxidation, solubility and reconstitution may affect technological properties and enzyme accessibility. However, effects on digestibility should be demonstrated experimentally rather than inferred solely from compositional or storage-related changes.

Usturoi et al. [11] compared whole egg powder packaged in high-density polyethylene (HDPE) and low-density polyethylene (LDPE) bags during 90 days of storage at 10 ± 0.5 °C and 65 ± 1% relative humidity. Moisture increased by 0.13 percentage points in the HDPE-packaged samples and by 0.95 percentage points in the LDPE-packaged samples. The LDPE-packaged powder also showed small numerical decreases in protein content, from 49.68% to 49.24%, and nitrogen-free extract, from 4.44% to 3.85%, whereas lipid and ash contents remained comparatively stable. These findings demonstrate that the barrier properties of the packaging material can influence the proximate composition of powdered egg during storage.

More recent evidence obtained under accelerated storage conditions showed packaging-related differences in moisture loss, caking, solubility, oxidative deterioration and thermal stability of whole-egg powder [12]. Aluminum-containing multilayer packaging provided better preservation of the evaluated quality attributes. These studies demonstrate effects on composition and product stability; corresponding changes in protein structure, enzyme accessibility or digestibility require direct experimental confirmation.

Powdered egg ingredients should therefore be evaluated across a sequence that includes drying, packaging, storage, rehydration, incorporation into the food product and digestion. Freshly prepared laboratory dispersions may not represent the composition or performance of ingredients after industrial storage. Application and shelf-life aspects of powdered egg ingredients are discussed further in Section 9.4.

### 8.5. In Vitro Digestion Models and Evidence Requirements

Standardized in vitro digestion models provide controlled conditions for comparing protein hydrolysis, peptide release and matrix effects. The INFOGEST static digestion protocol has improved methodological comparability, but it remains a simplified representation of human gastrointestinal digestion [31]. Static models do not fully reproduce gastric emptying, peristalsis, intestinal absorption, hormonal regulation, microbiota activity, inter-individual variability or long-term physiological adaptation.

Experimental conditions should be selected according to the intended product and consumer group. Complex foods containing lipids, polysaccharides, starch, salts or phenolic compounds may require careful interpretation because these components can affect enzyme activity and diffusion. Physiological differences between healthy adults and older individuals may also produce different digestion kinetics and peptide profiles [32,36,37].

Accordingly, in vitro digestion should be treated as one stage in an evidence pathway rather than as proof of bioavailability. Depending on the proposed function, evaluation may require post-digestion peptide profiling, intestinal transport studies, local intestinal-response models, safety and allergenicity assessment and confirmation in animals or humans. For functional food development, these biological assessments should be combined with technological performance, sensory acceptability, storage stability and validation in the intended food matrix.

### 8.6. Implications for Functional Food Development

For egg-derived proteins, functional food design should integrate technological performance with biological accessibility. A protein ingredient may be valuable because it forms gels, stabilizes emulsions or improves foam structure, but these properties may also affect digestion and peptide release. Similarly, a hydrolysate may show strong in vitro bioactivity but still be unsuitable for food application if it is unstable, bitter, poorly absorbed, allergenic or sensitive to storage conditions.

The key challenge is therefore to connect processing, matrix behavior and biological relevance. Egg-derived proteins should be evaluated not only as isolated molecules, but as ingredients that undergo processing, packaging, storage, food preparation, gastrointestinal digestion and intestinal absorption. This integrated approach is essential for translating promising egg-derived peptides and modified protein ingredients into functional foods with credible nutritional and biological value.

## 9. Techno-Functional Applications in Food Systems

The practical relevance of egg-derived proteins depends on their performance within complete food matrices and under conditions representative of manufacturing, distribution and storage. Egg white proteins provide foaming, gelation, water retention, film formation and, in some systems, emulsion stabilization. Egg yolk proteins, lipoproteins and phospholipids contribute mainly to emulsification, viscosity, lipid structuring and thermal stability. Mineral-binding capacity, although relevant to phosvitin and its derived phosphopeptides, is considered here a biological or nutritional function rather than a techno-functional property.

The modification strategies discussed in Section 4, Section 5 and Section 6 can alter these properties, but improvements measured in purified dispersions or model systems do not necessarily predict performance in foods. Sugars, lipids, salts, acidity, competing proteins, thermal processing and storage conditions may change protein behavior and may also affect flavor, color, texture and consumer acceptance. Application-oriented assessment should therefore combine instrumental functionality with product-specific processing, sensory and shelf-life measurements.

The principal application areas are summarized in Table 7. The table distinguishes the technological role of each egg-derived ingredient from the criteria used to assess its performance in food systems. It also indicates where evidence is based mainly on model systems and where validation in complete products remains necessary. This distinction is important because the degree of application-specific validation differs considerably among foamed products, emulsions, powders, beverages, delivery systems and protein-based materials.

The evidence summarized in Table 7 shows that no single modification strategy is appropriate for all food applications. Performance must be assessed against the requirements of the intended product. Aerated foods require stable gas–liquid interfaces and heat-set structures, emulsified foods require interfacial and oxidative stability, powders require preservation of functionality during storage and reconstitution, and beverages require solubility without unacceptable bitterness or sedimentation. Edible materials and non-edible food-contact systems require separate evaluation because they differ in intended use, consumer exposure, migration assessment and allergen-management requirements.

Accordingly, claims of improved techno-functional performance should be supported by tests conducted in the intended food matrix whenever possible. Studies limited to isolated protein systems remain useful for identifying mechanisms, but they cannot establish sensory acceptability, shelf-life, process compatibility or industrial feasibility. These application-specific considerations form the basis for the discussion in the following subsections.

### 9.1. Aerated Bakery and Confectionery Products

Egg white proteins are widely used as foaming and structure-forming ingredients in meringues, sponge cakes, angel cakes, macarons, nougat-type products and other aerated bakery and confectionery systems. Ovalbumin contributes to structure setting during heating, while ovomucin and the mixed albumen protein system support viscosity and foam stability. In these products, technological performance depends not only on initial foam volume, but also on the ability to retain air during mixing, deposition, baking or drying [2,6,97].

The functionality measured in protein dispersions cannot be transferred directly to complete formulations. Sugar can increase the viscosity of the continuous phase and delay foam drainage, but it can also modify protein unfolding and structure setting. Lipids may disrupt protein films at the air–water interface, while salts, acidity and interactions with flour or other formulation components may alter bubble stability. Consequently, modified egg white ingredients that perform well in standardized foaming assays may not produce comparable improvements in high-sugar, high-fat or thermally processed products.

Physical, enzymatic and chemical modification strategies have produced changes in the foaming behavior of egg white proteins [15,16,17,21,70,78,79,80,81]. However, their application value should be established using product-relevant measurements rather than interfacial or foam-capacity data alone. For bakery products, these measurements include batter density and stability, gas retention, baked volume, crumb-cell distribution, moisture retention, texture and staling behavior. For meringues and aerated confectionery products, relevant criteria include foam stability before processing, structural collapse during drying, fracture properties, hygroscopicity and crispness retention.

Sensory effects also require attention because treatments that modify protein behavior may influence color, egg-derived flavor, sweetness perception, crispness or crumb texture. Storage studies should evaluate moisture migration, structural collapse, textural changes and flavor stability under conditions appropriate to the intended product. From an industrial perspective, a modified egg white ingredient is useful only if its performance remains reproducible across batches and is compatible with conventional mixing, depositing, baking or drying operations. Current evidence supports the technological potential of modified egg white proteins, but further studies in complete food formulations are needed to determine whether improvements observed in model systems are maintained during processing and storage.

### 9.2. Gelled Foods, Desserts and Structured Matrices

Egg white proteins are used as gel-forming and structure-setting ingredients in custards, puddings, cooked egg products, protein-enriched desserts and other structured foods. Their technological contribution includes texture formation, water retention and stabilization of the product during processing and storage [34,35,36]. The required gel properties vary considerably among applications. Firm and elastic networks may be desirable in structured foods, whereas desserts, soft foods and products intended for older adults may require lower firmness, limited syneresis and appropriate swallowability.

Thermal processing remains the most established method for structuring egg protein gels, while high-pressure processing and transglutaminase treatment can provide different texture and microstructure profiles [65,66,68]. Glycation and succinylation have also been investigated for modifying water-holding capacity, elasticity and gel strength [79,83]. In this application-oriented context, the importance of these treatments lies in whether they produce a reproducible texture in the complete formulation rather than in their effects on isolated protein dispersions.

Assessment of gelled foods should include firmness, elasticity, fracture behavior, syneresis, water-holding capacity and stability during processing and refrigerated storage. Interactions with sugars, salts, lipids, hydrocolloids and other proteins may alter the gel behavior observed in simplified systems. Sensory evaluation is also required because increased instrumental gel strength may be accompanied by undesirable rubberiness, dryness, graininess or reduced flavor release.

Digestibility should be evaluated independently rather than inferred from gel strength or cross-link density. Dense or extensively cross-linked structures may restrict digestive-enzyme accessibility, but direct evidence demonstrating reduced digestibility in transglutaminase-treated egg protein foods remains limited. Product development should therefore assess texture, rheology, microstructure and gastrointestinal behavior together when nutritional performance is part of the intended application.

Industrial suitability also depends on batch reproducibility, compatibility with conventional heating or pressure-processing equipment, processing time and stability throughout the intended shelf-life. Current evidence demonstrates the capacity of egg-derived proteins to form gels with different structural characteristics, but further studies in complete food products are needed to establish the balance among texture, sensory acceptability, storage stability and digestibility.

### 9.3. Emulsion-Based Foods, Sauces and Dressings

Egg yolk is an established emulsifying ingredient in mayonnaise, sauces, dressings, creams, fillings and other oil-in-water emulsions. Its performance is associated mainly with low-density lipoproteins, phospholipids and other protein–lipid assemblies that stabilize dispersed oil droplets [3,5,56]. In complete food formulations, egg yolk also contributes to viscosity, mouthfeel, color and flavor, making its technological role broader than emulsion stabilization alone.

The suitability of an egg yolk ingredient depends on its behavior under the composition and processing conditions of the intended product. Oil fraction, pH, salt concentration, homogenization intensity and thermal treatment can affect droplet formation, viscosity and physical stability. Processing and storage of egg yolk powders may also alter emulsifying performance, oxidative stability and flavor quality [98]. Consequently, results obtained from freshly prepared model emulsions cannot fully predict performance in acidic sauces, high-oil formulations or products subjected to pasteurization and prolonged storage.

Selected modification strategies have been investigated to address specific application limitations. Phospholipase-treated yolk systems have shown changes in emulsifying capacity and thermal stability that may be relevant to heat-processed sauces and dressings [62]. Succinylation of egg yolk low-density lipoproteins has been associated with reduced thermal oxidation–aggregation and off-flavor formation in lipid-rich systems [84]. Short-term fermentation followed by post-treatment has also been evaluated in relation to the functionality of egg yolk ingredients used in powdered oil systems [99]. These findings indicate potential application benefits, but comparisons remain difficult when studies use different formulations, processing conditions and stability criteria.

Evaluation in actual products should combine droplet-size distribution, creaming or phase separation, rheological behavior and stability during heating with measurements of lipid oxidation and flavor development. Color, mouthfeel, acidity perception and consumer acceptance are also relevant because modification may affect both the technological performance and the sensory identity traditionally associated with egg yolk-containing foods.

Industrial implementation requires ingredients that retain their emulsifying capacity after drying, transport, reconstitution and storage and that remain compatible with existing homogenization and thermal processing operations. Batch consistency, susceptibility to oxidation, off-flavor development and storage temperature should therefore be assessed over the intended shelf-life. Further product-level studies are needed to determine whether the benefits reported in model systems are retained in commercial-type sauces, dressings and other emulsion-based foods.

### 9.4. Powdered Egg Ingredients and Dry Food Systems

Whole egg powder, egg white powder and egg yolk powder are used in bakery products, confectionery, sauces, desserts, instant mixes and protein-enriched formulations. Compared with liquid egg ingredients, powders offer longer storage, lower transport and refrigeration requirements, easier dosing and compatibility with continuous industrial production. These advantages depend, however, on maintaining the required functionality during drying, storage, reconstitution and subsequent food processing [11,12,98,100].

Egg powders cannot be regarded as direct functional equivalents of their liquid counterparts. Drying and storage may reduce solubility, dispersibility, foaming, emulsification or gelation performance and may also produce caking, color changes, lipid oxidation and off-flavors [11,12,98,100]. The effects differ among egg white, yolk and whole egg powders because their protein, lipid and carbohydrate compositions create different stability requirements. Egg yolk and whole egg powders require particular control of lipid oxidation, whereas egg white powders must retain the foaming or gelation properties required by the intended formulation.

Spray-drying conditions have been associated with changes in the gelation behavior of egg white proteins [72], while the drying method can influence the functional and antioxidant properties of egg-derived peptide powders [73]. Fermentation before spray-drying has also been investigated for increasing the solubility, dispersibility and simulated digestibility of egg yolk powder [63]. The practical importance of these findings depends on whether the reported properties remain stable during storage and are recovered after rehydration under industrially relevant conditions.

Product-specific evaluation should include moisture content, water activity, particle-size distribution, bulk density, flowability, caking tendency, wettability, dispersibility and solubility. After reconstitution, the relevant application properties should also be measured. These may include foam formation and stability for bakery or confectionery products, emulsion stability for sauces and dressings, and gel strength and water retention for structured foods. Testing immediately after drying is insufficient when the ingredient is intended for extended storage.

Packaging conditions are particularly important because moisture and oxygen exposure may accelerate caking, oxidation, flavor deterioration and loss of functionality. Storage studies should therefore consider packaging barrier properties, storage temperature, relative humidity, light exposure and the intended shelf-life. Sensory assessment after reconstitution is also necessary because acceptable powder flow and solubility do not exclude oxidized, sulfurous or other undesirable flavor notes.

Industrial adoption depends on consistent powder quality, reliable rehydration, compatibility with existing mixing and processing equipment and retention of functionality throughout storage. Evidence obtained from fresh laboratory powders should therefore be complemented by application trials using stored ingredients in complete food formulations. Such testing is necessary to establish whether the technological advantages of powdered egg ingredients are retained under realistic production and distribution conditions.

### 9.5. Protein-Enriched Beverages and Liquid Food Systems

Soluble egg white fractions and egg protein hydrolysates may be incorporated into protein-enriched beverages, liquid nutritional products and formulations intended for older adults, physically active consumers or individuals with specific nutritional requirements. In these products, egg-derived ingredients can contribute high-quality protein and, depending on the preparation, peptide fractions with potential biological relevance. Their suitability for beverages depends primarily on dispersion stability, sensory compatibility and resistance to processing and storage conditions.

Beverage formulations impose requirements that are not adequately represented by solubility measurements alone. Protein ingredients must remain dispersed across the intended pH range and during homogenization, pasteurization or other thermal treatments. Turbidity, sedimentation, viscosity and aggregation must remain acceptable throughout storage. Interactions with minerals, sugars, acids, flavor compounds and other proteins can also alter the behavior observed in simplified aqueous systems.

Hydrolysis, pH adjustment and other modification strategies have been investigated for obtaining more dispersible egg protein ingredients [15,16,17,20,58]. However, hydrolysis may produce bitterness or persistent aftertastes, while some modified fractions may remain sensitive to heat, acidity or prolonged storage [49]. Product development should therefore combine measurements of solubility, particle size, turbidity and sedimentation with sensory assessment of flavor, mouthfeel and appearance. Shelf-life studies should also determine whether initially stable beverages develop aggregation, phase separation or undesirable flavor changes during storage.

Antioxidant or ACE-inhibitory activity measured in isolated hydrolysates should be distinguished from the nutritional function of the beverage. Such in vitro findings do not establish peptide bioavailability or a physiological effect after consumption. Gastrointestinal stability, intestinal absorption and effective dose remain uncertain for many egg-derived peptide mixtures [29,30,31,32,33,94]. Any health-related positioning would therefore require evidence appropriate to the intended claim and target population.

Commercial application also requires reproducible ingredient composition, compatibility with existing beverage-processing equipment and clear allergen information. The use of egg-derived protein in a beverage must be considered in relation to consumer expectations, because egg flavor, visible turbidity or allergen concerns may reduce acceptance even when nutritional and physical-stability criteria are met. Further studies in complete beverage formulations are needed to establish acceptable incorporation levels and stability during processing and storage.

### 9.6. Egg-Protein-Based Edible Materials and Food-Contact Packaging

Egg white proteins can be used to produce continuous films or composite materials with structural, barrier or active properties [101]. However, edible films and coatings should be distinguished from non-edible packaging and other food-contact materials. The two categories differ in intended use, consumer exposure, performance requirements and regulatory assessment. Edible materials are designed to remain on the product and be consumed with it, whereas packaging and food-contact materials are not intended for ingestion.

#### 9.6.1. Edible Films and Coatings

Edible egg-protein-based films and coatings may provide mechanical protection, reduce gas transfer or serve as carriers of antioxidant and antimicrobial compounds. Their performance on food depends on adhesion, film continuity, flexibility, transparency and resistance to changes in temperature and relative humidity. Oxygen and water-vapor permeability are particularly relevant because the hydrophilic nature of protein-based materials may limit their performance under high-humidity conditions.

Egg white protein–polysaccharide and protein–polyphenol combinations have been investigated to modify the mechanical, barrier and active properties of these materials [85,86,101]. Egg white protein–pullulan films containing pomegranate peel polyphenols have also been evaluated for berry fruit preservation, providing an example of application in a food product rather than characterization of the film alone [102]. Such studies should report not only mechanical and antioxidant properties but also changes in fruit quality, microbial stability, appearance and storage life.

Since edible films and coatings are consumed with the food, sensory compatibility is essential. Thickness, surface appearance, mouthfeel, taste and odor may determine whether a technically effective coating is acceptable to consumers. The composition must also be suitable for ingestion, and the presence of egg-derived proteins requires appropriate allergen management and information in accordance with the requirements applicable to the marketed product and jurisdiction.

#### 9.6.2. Active Packaging and Other Food-Contact Materials

Egg-protein-containing packaging films, active layers or other food-contact materials that are not intended for consumption require a different assessment framework. Their relevant properties include mechanical integrity, barrier performance, stability of the active component and maintenance of antimicrobial or antioxidant activity during the intended period of use. Performance should be evaluated with the packaged food because moisture, fat, acidity and contact duration may alter the behavior of the material.

Migration and transfer to food must be assessed when egg-derived proteins, peptides, polyphenols or other active substances are incorporated into a food-contact material. Material characterization alone cannot establish suitability for food-contact use. Testing should consider the intended food category, temperature, contact time, storage conditions and applicable migration and safety requirements.

The use of egg-derived proteins in non-edible food-contact materials may also require allergen-risk assessment if transfer to the food is possible. The regulatory and labeling consequences cannot be generalized because they depend on material composition, intended use, migration behavior and jurisdiction. These factors should therefore be evaluated separately from the requirements applicable to edible films and coatings.

### 9.7. Delivery Systems for Bioactive Compounds and Nutraceutical Ingredients

Egg white proteins, egg yolk lipoproteins and lecithin-containing fractions can serve as components of delivery systems for phenolic compounds, vitamins, pigments, lipophilic substances, peptides and other bioactive ingredients [6,7,8,9]. Egg white proteins are suitable for gelled or particulate carriers, while yolk-derived lipoproteins and phospholipids can support emulsion- or lipid-based systems. Their value in food applications depends on their ability to retain the incorporated compound during processing and storage and to release it under the intended gastrointestinal conditions.

Protein–polysaccharide and protein–polyphenol combinations have been studied as methods of modifying colloidal stability and incorporating antioxidant compounds [81,85,86]. In application-oriented assessment, encapsulation efficiency alone is insufficient. Delivery systems should also be evaluated for resistance to heat, changes in pH and ionic strength, oxidation, dilution and interactions with other food components. Loss of the incorporated compound or aggregation of the carrier during processing may eliminate benefits measured immediately after preparation.

Testing in complete foods is particularly important because the carrier can affect color, flavor, turbidity, viscosity and texture. An emulsion- or particle-based system that remains stable in a laboratory dispersion may destabilize after incorporation into a beverage, dessert, sauce or other multicomponent product. Sensory effects should therefore be assessed alongside physical stability and retention of the encapsulated compound throughout storage.

The biological relevance of a delivery system depends on release, bioaccessibility and, where applicable, intestinal transport rather than only on the amount initially encapsulated. Simulated gastrointestinal digestion can provide information on release and stability, but it does not replace evidence of absorption or physiological activity. Egg-derived delivery systems also retain the allergenic relevance of their protein source, which must be considered during formulation, safety assessment and consumer communication.

Industrial use will require reproducible carrier composition, scalable preparation, stability during distribution and compatibility with standard food-processing operations. Current findings support the technical feasibility of egg-derived delivery structures, but evidence obtained in simplified systems should be complemented by studies in complete foods and under realistic processing, storage and digestion conditions.

### 9.8. Comparative Application and Industrial Perspective

The suitability of an egg-derived protein ingredient is determined by the requirements of the intended food rather than by improvement in a single laboratory parameter. Egg white proteins are principally suited to foamed, gelled and film-forming systems, whereas egg yolk lipoproteins and phospholipids are more directly applicable to emulsified foods and lipid-based delivery systems. Hydrolysates and peptide-rich fractions may be incorporated into beverages or other protein-enriched products, but bitterness, compositional variability, gastrointestinal stability and uncertain bioavailability remain important limitations.

The strength of the available evidence differs among applications. Native egg white and yolk ingredients have established roles in bakery products, confectionery, sauces and dressings. In contrast, many modified protein preparations, peptide delivery systems and protein-based films have been studied, mainly in isolated dispersions, model emulsions, laboratory gels or material-characterization experiments. These studies identify technological possibilities but do not demonstrate performance in commercial-type foods.

Product-oriented validation should assess four connected dimensions: performance during processing, stability during storage, sensory acceptability and compatibility with nutritional or biological objectives. A modification that increases foam capacity but reduces baked-product stability, or strengthens a gel while producing an unacceptable texture, does not provide an application benefit. Similarly, a hydrolysate with biological activity measured in vitro may have limited value if bitterness prevents incorporation at an effective concentration.

Industrial translation also depends on batch-to-batch reproducibility, ingredient standardization, processing time, water and energy requirements, production cost and compatibility with existing equipment. Egg-allergen management applies across applications, but the relevant measures differ among foods, edible materials and non-edible food-contact systems. Regulatory assessment must therefore consider the composition, intended use, consumer exposure and jurisdiction applicable to each product category.

The main evidence gap remains the limited number of studies that follow modified egg-derived ingredients through complete product development, including manufacturing, storage, sensory evaluation and, when biological effects are proposed, digestion or human validation. Future studies should identify the food matrix and intended technological function at the beginning of ingredient development and should use application-specific benchmarks. This approach would permit a more reliable assessment of whether a modified egg-derived protein can function as a stable, acceptable and reproducible food ingredient under industrially relevant conditions.

## 10. Safety, Allergenicity, Sensory Quality and Regulatory Considerations

The development of egg-derived functional ingredients requires simultaneous consideration of technological performance, allergenicity, sensory quality, storage stability and regulatory acceptability. Hydrolysis, fermentation and physical, chemical or biochemical treatments can modify protein structure, peptide composition and matrix interactions, with corresponding effects on IgE binding, digestibility, flavor, color and ingredient identity. These outcomes are process-, protein- and matrix-specific and should be evaluated for the intended application.

Egg white contains several clinically relevant allergens, including ovomucoid, ovalbumin, ovotransferrin and lysozyme, and egg yolk allergens may also be relevant in products containing whole egg, yolk or isolated yolk fractions [42,43,44,45,46,47]. Processing can alter conformational or linear epitopes, aggregation and digestion resistance, but cannot be assumed to eliminate allergenic potential [42,43,44,45,46,47,103]. Modified egg proteins should therefore not be described as hypoallergenic without appropriate analytical, immunological and clinical evidence. Table 8 summarizes the principal safety, sensory and regulatory considerations.

### 10.1. Allergenicity and Processing-Dependent Effects

Ovomucoid is particularly relevant to allergenicity assessment because of its relative heat stability, protease resistance and persistence during digestion [42,43,44,45,46,47,104]. Ovalbumin, ovotransferrin and lysozyme also require consideration because their structures and immunoreactivity may change during processing. Egg yolk allergens should be included when evaluating whole egg, yolk powder, lipoprotein fractions or yolk-based delivery systems.

Processing may destroy conformational epitopes, mask selected binding sites, promote aggregation or increase susceptibility to digestion. Conversely, it may expose previously inaccessible epitopes, preserve digestion-resistant fragments or generate new antigenic structures [42,43,44,45,46,47,103]. Reviews of glycation, ultrasound, pulsed electric fields, irradiation, high-pressure processing and cold plasma confirm that the direction and magnitude of these effects vary with the allergen and treatment conditions [103]. Allergenicity reduction must therefore be demonstrated for the specific protein preparation and process rather than attributed generally to a modification technology.

Traditional cooking methods, including boiling, steaming, baking and frying, reduced immunological responses under selected experimental conditions evaluated through simulated digestion, serological assays, cell degranulation and animal models [105]. Controlled glycation of structurally modified ovomucoid also reduced IgE- and IgG-binding capacity in a specific experimental system [104]. These findings demonstrate treatment-dependent changes in immunoreactivity but do not establish clinical tolerance. In addition, reduced IgE binding before digestion may not predict the immunoreactivity of the fragments subsequently released. Modified ingredients should therefore be evaluated both before and after digestion, with clinical interpretation restricted to evidence obtained using appropriate human studies.

### 10.2. Analytical Control of Egg Allergens

Processing can affect allergen detection by changing protein extractability, aggregation, peptide composition and antibody recognition. This is especially important for hydrolyzed, glycated or conjugated ingredients, for which methods developed for native egg proteins may provide incomplete results.

Multi-allergen liquid chromatography–tandem mass spectrometry (LC–MS/MS) methods can quantify several major egg allergens, including Gal d 1–6, in processed food matrices [47,106]. Such methods reduce reliance on a single marker protein, but analytical concentration alone does not define clinical risk. Protein structure, digestion stability, consumed dose, matrix composition and individual sensitivity must also be considered. Protein profiling, peptide mapping, allergen quantification, simulated digestion and IgE-binding assays should therefore be regarded as complementary components of the safety assessment.

### 10.3. Sensory Quality and Consumer Acceptance

Sensory quality is a practical limitation for egg-protein hydrolysates, peptide fractions and modified powders. Enzymatic hydrolysis may generate hydrophobic peptides associated with bitterness, whereas fermentation, lipid oxidation, Maillard reactions and storage can produce sour, sulfurous, metallic, rancid or stale notes [49,98,107,108]. Sensory effects should therefore be evaluated during process development rather than only in the final formulation.

Enzyme selection, control of hydrolysis degree, membrane fractionation, fermentation, encapsulation, flavor masking and antioxidant protection may reduce undesirable sensory characteristics [49,107,108]. However, these interventions may also alter peptide composition, biological activity, functionality or ingredient labeling. For example, removing a bitter fraction may also remove potentially active peptides. Sensory optimization should consequently be assessed together with composition, oxidation stability and performance in the intended food product.

### 10.4. Clean-Label, Allergen Labeling and Health-Claim Considerations

The term “clean-label” is used here as a consumer- and market-oriented concept rather than as a uniform regulatory classification. It may encompass ingredient familiarity, formulation simplicity, process transparency, sensory expectations and perceptions of processing. Clean-label compatibility therefore cannot be inferred solely from whether a treatment is physical, enzymatic, fermentation-assisted or chemical.

Physical, enzymatic and fermentation-assisted processes may support clean-label formulation when they reduce the need for additional additives. Their acceptability nevertheless depends on the enzymes or microorganisms used, processing aids, resulting composition, ingredient designation and consumer expectations. Chemical modifications such as succinylation, phosphorylation and selected conjugation reactions require assessment of reagent specifications, reaction products, residual compounds, food-grade status and the regulatory identity of the modified ingredient. No treatment category should be considered inherently compatible or incompatible with clean-label expectations.

In the European Union, Regulation (EU) No. 1169/2011 identifies eggs and products thereof as substances or products causing allergies or intolerances that must be declared to consumers [48]. Hydrolysis, fractionation, modification or incorporation into a delivery system should not be assumed to remove this obligation. The applicable allergen declaration and safety requirements must be determined for the specific ingredient, manufacturing process and final food.

Experimental antioxidant, ACE-inhibitory, antimicrobial or anti-inflammatory activity does not, by itself, substantiate a consumer-facing health claim. Claims regarding physiological benefits, reduced allergenicity or improved tolerance require evidence appropriate to the specific ingredient and intended use and must comply with the requirements of the jurisdiction in which the product is marketed. Regulatory assessment should therefore address ingredient identity, production process, compositional changes, allergen management, intended use and claim substantiation from the beginning of product development.

### 10.5. Integrated Safety Assessment

Safety assessment should be adapted to the ingredient and its intended application. An egg white hydrolysate intended for beverages requires evaluation of peptide composition, residual allergenicity, bitterness, heat stability and shelf-life. A modified yolk ingredient intended for emulsified foods requires assessment of allergen content, lipid oxidation, flavor stability and performance during storage. Accordingly, the development pathway should combine structural characterization, validated allergen analysis, digestion testing, sensory evaluation, storage studies, regulatory review and validation in the intended food matrix. This integrated approach allows technological performance to be considered together with allergenic risk, sensory acceptability and regulatory feasibility.

## 11. Industrial Scalability, Sustainability and Future Perspectives

The scientific potential of egg-derived proteins as functional ingredients is well supported by studies on structural modification, bioactive peptide release, digestibility and techno-functional performance. However, the transition from laboratory-scale experiments to industrially relevant food ingredients remains incomplete. Many promising results are still obtained in purified protein dispersions, model emulsions, simplified gels or small-volume hydrolysate systems. Although such studies are essential for understanding mechanisms, they do not necessarily predict performance during industrial processing, long-term storage, formulation in complex foods or consumer use. Therefore, industrial scalability, sustainability and application-oriented validation must be considered as central criteria in the future development of egg-derived functional ingredients.

The main challenge is that egg-derived proteins are structurally complex and matrix-sensitive. Egg white proteins, yolk lipoproteins, phosvitin-containing granules, whole egg powders and hydrolysate fractions respond differently to heat, pressure, ultrasound, drying, fermentation, chemical modification and gastrointestinal digestion. Their final functionality depends not only on the selected modification strategy, but also on raw material variability, pretreatment, pH, ionic strength, water activity, lipid oxidation, enzyme specificity, reaction time, drying conditions, packaging and storage. As discussed in previous sections, a modification that improves solubility, foaming or peptide release may simultaneously reduce gel strength, increase bitterness, alter allergenicity or impair sensory quality. For this reason, the industrial development of egg-derived functional ingredients requires integrated optimization rather than single-parameter improvement.

The key barriers and future research priorities for the industrial translation of egg-derived proteins are summarized in Table 9. These priorities reflect the need to connect molecular characterization with process control, food-grade feasibility, sensory acceptability, safety assessment, storage stability and validation in realistic food systems.

### 11.1. From Laboratory Modification to Industrial Processing

Laboratory studies provide essential mechanistic insight into how egg-derived proteins respond to enzymatic hydrolysis, fermentation, high-pressure processing, ultrasound, cold plasma, pH-shifting, glycation, phosphorylation or succinylation. However, industrial translation requires more than proof of structural change or improved functionality under controlled small-scale conditions. Industrial processes must tolerate raw material variability, larger batch volumes, continuous or semi-continuous operation, equipment limitations, cleaning requirements, energy consumption, water use, cost constraints and regulatory expectations.

This issue is particularly important for egg protein hydrolysates. Enzymatic food protein hydrolysates are increasingly recognized as multifunctional ingredients with improved solubility, interfacial behavior and potential bioactivity, but their production requires strict control of enzyme specificity, degree of hydrolysis, reaction conditions, bitterness and downstream processing [109]. For egg-derived hydrolysates, the same principles apply. A hydrolysis process that performs well at bench scale may generate different peptide profiles when scaled up, especially if temperature distribution, pH control, enzyme-to-substrate ratio, mixing efficiency or inactivation conditions change. Therefore, future egg-protein hydrolysate studies should report process parameters in sufficient detail to support reproducibility and scale-up.

Physical and fermentation-assisted technologies face similar challenges. Ultrasound treatment may improve foaming or solubility at laboratory scale, but industrial application requires uniform energy distribution and controlled cavitation in larger volumes [17]. High-pressure processing may generate desirable gels or improve texture, but cost, throughput and product-specific optimization remain limiting factors [68]. Fermentation-assisted modification may improve solubility or digestibility, but strain selection, pH evolution, metabolite formation and drying conditions must be standardized [20,63]. Therefore, process scalability should be assessed as an experimental outcome, not only discussed as a future possibility.

### 11.2. Standardization of Peptide-Rich Ingredients

Peptide standardization is one of the most important requirements for translating egg-derived peptides into functional foods. Hydrolysates are complex mixtures containing peptides of different sizes, sequences, charges, hydrophobicities and digestion stabilities. Their activity may result from a combination of peptides rather than from a single sequence. This complexity makes it difficult to compare studies, define active ingredients, establish quality specifications or support biological claims.

Recent reviews on food protein hydrolysates and bioactive peptides emphasize the need to connect hydrolysis conditions with molecular-weight distribution, sequence identification, structure–activity relationships, bioavailability and functional performance [109,110]. In egg-derived systems, batch-controlled hydrolysis and peptide profiling are especially important because peptide composition may vary with protein source, enzyme type, reaction time, substrate pretreatment and downstream processing [58]. Fractionation methods such as ultrafiltration, membrane separation and chromatographic purification can enrich specific peptide fractions, but they also introduce cost, yield loss and scalability challenges [111].

A practical future direction would be to define ingredient specifications for egg-derived peptide preparations. These may include protein source, degree of hydrolysis, peptide-size distribution, marker peptides, residual allergen content, bitterness score, solubility, bioactivity assay, digestion stability and storage performance. Without such specifications, egg-derived peptide ingredients will remain difficult to reproduce and difficult to translate into commercial functional foods.

### 11.3. Food-Grade Feasibility and Clean-Label Considerations

Food-grade feasibility and clean-label compatibility should be treated as related but distinct considerations in the development of modified egg-derived ingredients. A process may use food-grade materials and comply with applicable safety requirements without necessarily meeting consumer expectations associated with a clean-label product. Conversely, the absence of synthetic chemical additives does not by itself establish process transparency, consumer acceptance or suitability for clean-label positioning.

Enzymatic hydrolysis, fermentation and physical treatments such as ultrasound, high-pressure processing and controlled drying may support certain clean-label formulation strategies because they can be applied through established food-processing operations. However, these treatments should not be described as inherently clean-label. Their acceptability depends on the enzymes or microbial cultures used, processing aids, resulting ingredient identity, degree of processing, compositional changes, sensory effects and the way in which the process is communicated to consumers. pH-shifting and glycation with food-compatible carbohydrates require the same case-specific assessment [17,18,19,20,21,22,23,63,68,78,79,80,81,82,83,84,85,86,103,112].

Chemical modifications, including succinylation, phosphorylation and selected conjugation reactions, remain valuable for studying protein structure–function relationships and may also have specialized technological applications. Their transfer to food production requires assessment of reagent specifications, reaction by-products, residual compounds, processing aids, ingredient identity and applicable regulatory requirements. These considerations should be included during process development rather than evaluated only after the desired functional property has been obtained.

Future studies should therefore distinguish among experimental methods used for mechanistic investigation, food-grade processes suitable for ingredient production and processes that may also meet market-specific clean-label expectations. Evaluation should include process reproducibility, scalability, energy and water requirements, ingredient standardization, sensory quality and stability throughout the intended shelf-life. For egg-derived proteins, allergen management remains necessary regardless of whether the modification is enzymatic, fermentation-assisted, physical or chemical.

Clean-label translation should consequently be considered at the level of the complete product rather than attributed to a processing method in isolation. Ingredient naming, formulation complexity, process transparency, sensory familiarity, documented safety and consumer perception all influence clean-label positioning. Product development should combine these considerations with allergen management, storage studies and validation in realistic food matrices.

### 11.4. Environmental Considerations and Protein-Focused By-Product Valorization

Egg-processing side streams may provide additional sources of proteins and peptides for functional ingredient development. Relevant materials include salted egg white, defatted egg yolk proteins and protein-containing fractions recovered during the manufacture of liquid or powdered egg products [88]. Their valorization may reduce the amount of material treated as low-value residue and may increase the recovery of food-grade components from existing processing streams. However, waste reduction or recovery of a useful ingredient should not, by itself, be interpreted as evidence of environmental sustainability.

The environmental performance of a recovery or modification process depends on several connected factors, including extraction yield, energy and water consumption, enzymes, solvents or other processing inputs, purification requirements, effluent generation and the stability of the recovered ingredient. Additional drying, concentration or fractionation stages may require substantial energy and may offset some of the benefits associated with material recovery. The location and scale of processing, transport requirements and integration with existing egg-processing operations may also affect environmental performance. In the absence of comparative process-level data or life-cycle assessment, the environmental benefits attributed to egg-protein valorization should therefore be regarded as hypothetical rather than demonstrated.

Egg-processing by-products have been investigated as sources of peptides with antioxidant, anti-inflammatory, metal-chelating and other potential biological activities [88]. For protein-focused valorization, future studies should extend beyond extraction yield or activity measured in vitro. Relevant criteria include protein or peptide recovery, batch consistency, food-grade processing, gastrointestinal stability, safety, sensory effects, shelf-life and performance in complete food products. Economic feasibility should also be considered because low recovery yields, intensive purification or high processing costs may prevent industrial implementation even when laboratory results are favorable.

Improving the storage stability and rehydration of egg white, egg yolk and whole egg powders may also contribute to more efficient use of processed egg ingredients [98,100]. Longer functional shelf-life and reduced deterioration may limit product rejection or loss during distribution. Nevertheless, these benefits should be described as potential contributions to resource efficiency rather than as demonstrated reductions in environmental impact unless they are supported by appropriate comparative data.

Life-cycle assessment, carbon-footprint analysis, water-use assessment or other environmental evaluation methods can provide a more complete comparison between valorization routes and conventional disposal or ingredient-production systems. Such assessments should define system boundaries, energy sources, allocation procedures, processing scale and the reference product used for comparison. Where quantitative environmental data are unavailable, sustainability claims should remain qualified and should distinguish demonstrated material recovery from inferred environmental benefits.

Eggshell membrane, eggshell-associated proteins, minerals, glycosaminoglycans and other egg-processing fractions may also have valorization potential [88]. However, components that are not protein- or peptide-based extend beyond the central scope of this review. They are therefore acknowledged only as part of the broader context of egg-processing by-product utilization, while this discussion remains focused on egg-derived protein ingredients and peptide systems.

### 11.5. Translational Research Priorities

Future research should adopt application-oriented designs that connect protein source, modification conditions, molecular characteristics and performance in the intended food product. Priority should be given to standardized production of peptide-rich and modified protein ingredients, including marker peptides, molecular-weight distributions, process specifications and batch-reproducibility criteria [58,109,110,111]. Pilot-scale studies should evaluate ingredient performance after processing and realistic storage in complete food matrices rather than relying primarily on freshly prepared laboratory dispersions. The digestion, bioavailability, safety, allergenicity and sensory requirements described in Section 7, Section 8 and Section 10 should be incorporated according to the intended function and consumer group.

Computational approaches, including in silico screening, peptide databases, molecular docking, network pharmacology and machine-learning tools, may support peptide selection and process optimization [58,110,112]. These methods should be used to generate and prioritize hypotheses rather than as substitutes for experimental validation. Translation into food applications ultimately requires reproducible processing, defined composition, acceptable sensory properties, storage stability, regulatory feasibility and demonstrated performance in the intended product. Where a biological benefit is proposed, the supporting evidence should progress from mechanistic screening to digestion and transport assessment and, when justified, controlled human studies.

## 12. Conclusions

Egg-derived proteins provide complementary technological and nutritional functions relevant to food formulation. Egg white proteins contribute mainly to foaming, gelation, water retention and film formation, whereas yolk proteins, lipoproteins and phospholipids support emulsification, viscosity control and lipid structuring. Egg proteins can also serve as sources of amino acids, bioactive peptides and mineral-binding phosphopeptides.

Enzymatic, fermentation-assisted, physical, chemical and biochemical treatments can modify these properties, but their effects depend on the protein fraction, processing conditions and food matrix. Techno-functional improvement should be distinguished from biological or nutritional relevance. Most evidence for the reported antioxidant, anti-inflammatory, antihypertensive, antimicrobial and immunomodulatory activities remains preclinical, and clinical efficacy cannot be inferred without evidence of gastrointestinal stability, absorption, effective dose and responses in humans.

Translation into food products requires simultaneous consideration of functionality, allergenicity, sensory quality, storage stability and regulatory acceptability. Processing should not be assumed to eliminate egg-protein immunoreactivity, and food-grade feasibility does not automatically establish clean-label compatibility. Ingredient identity, processing aids, process transparency, appropriate allergen labeling and consumer acceptance must be evaluated for the intended product and market.

Future studies should prioritize standardized ingredient production, batch reproducibility, defined molecular composition and validation at pilot scale in complete food matrices. Digestion and transport assessment, allergenicity after processing and digestion, sensory evaluation and shelf-life testing should be selected according to the proposed application. Claims of environmental benefit also require evidence concerning energy and water use, processing inputs, economic feasibility and, where available, comparative environmental assessment. Egg-derived functional ingredients will be suitable for industrial application only when their technological performance, safety, nutritional relevance and product stability are demonstrated within the same development framework.

## Figures and Tables

**Figure 1:**
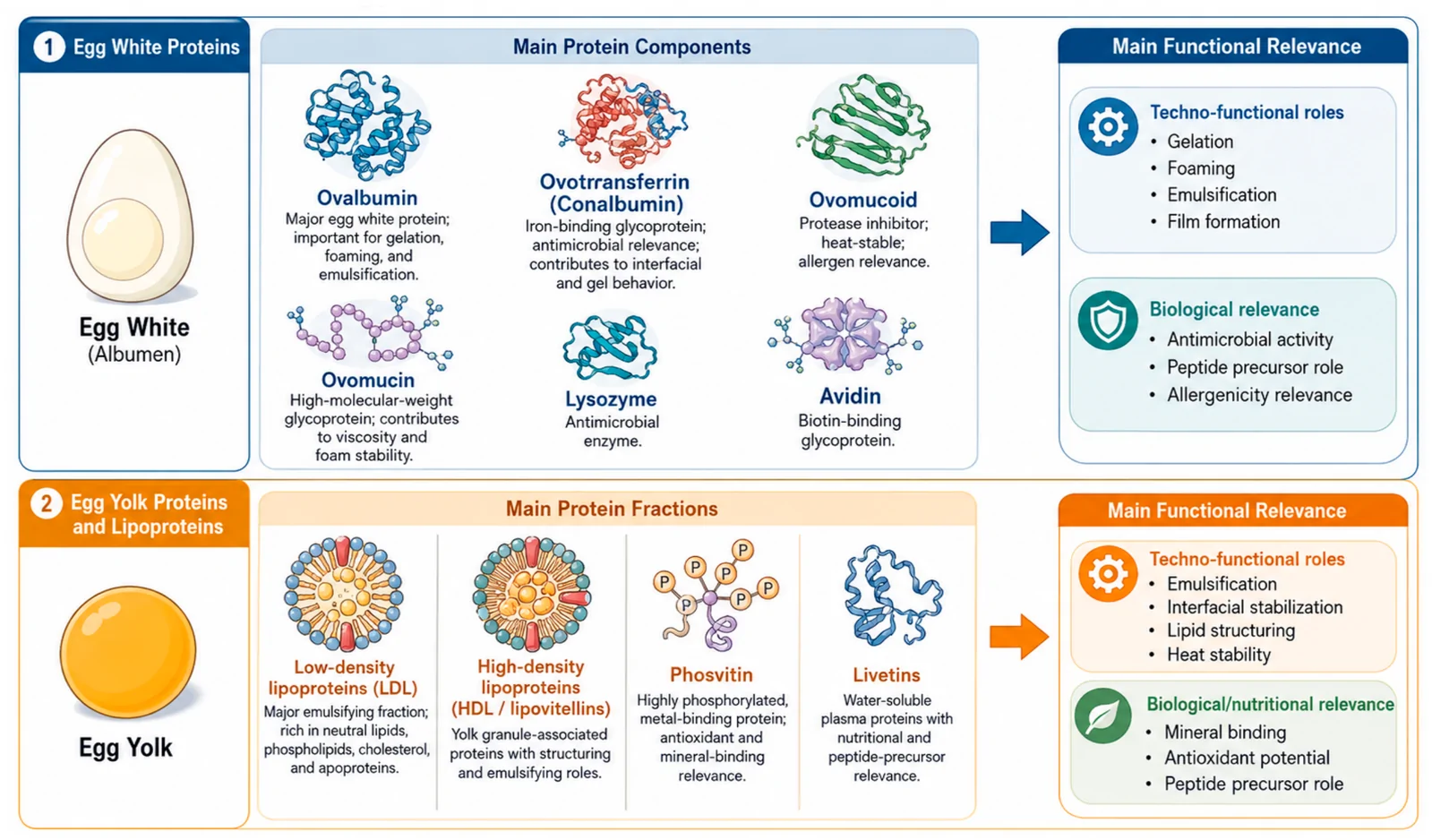
Native egg-derived proteins and their structure–function relevance. Conceptual overview linking the major egg white and egg yolk protein components with their principal techno-functional and biological or nutritional roles.

**Figure 2:**
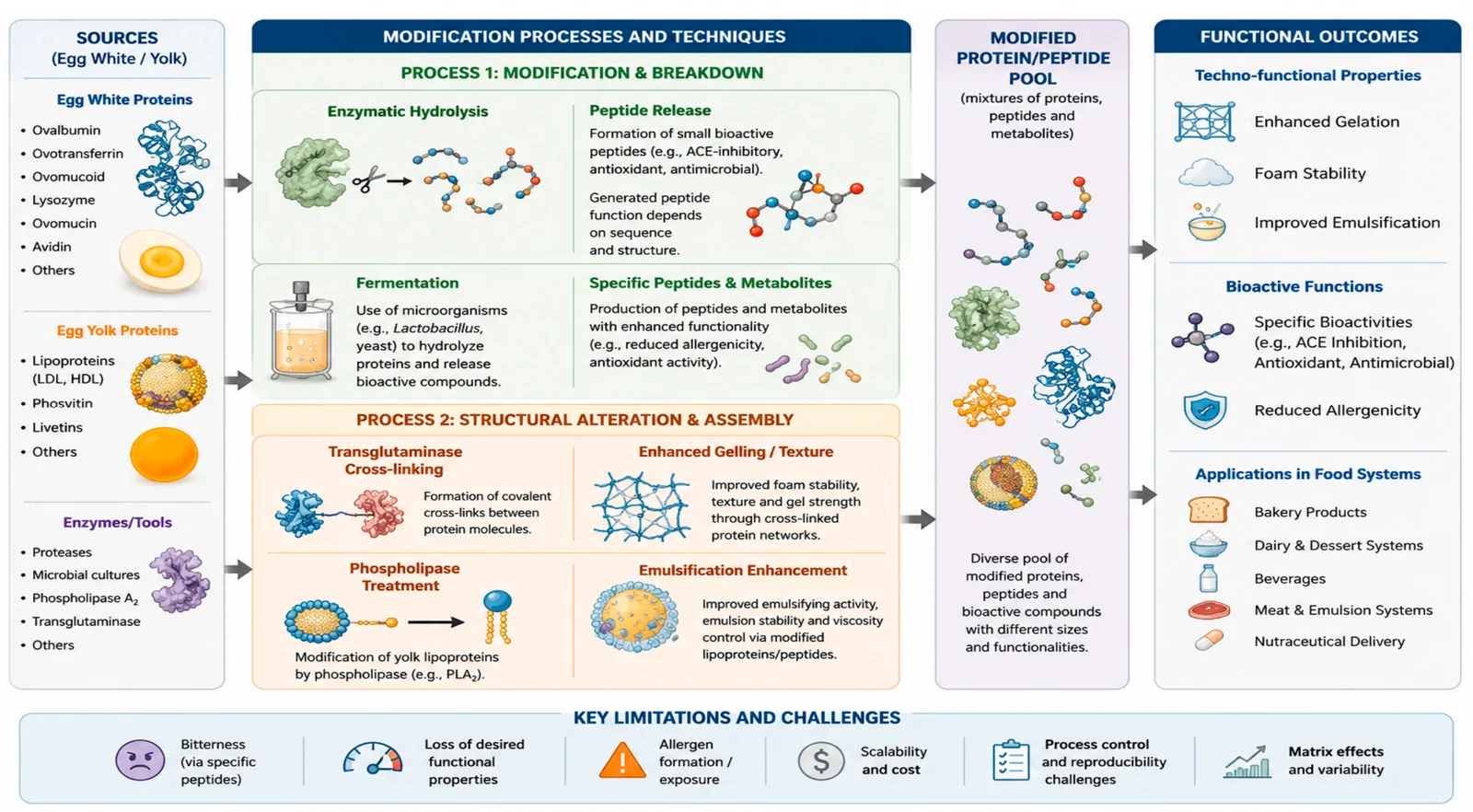
Enzymatic and fermentation-assisted modification of egg-derived proteins. Schematic representation of proteolytic hydrolysis, fermentation, transglutaminase-mediated cross-linking and phospholipase treatment and their principal effects on egg white and egg yolk protein systems.

**Figure 3:**
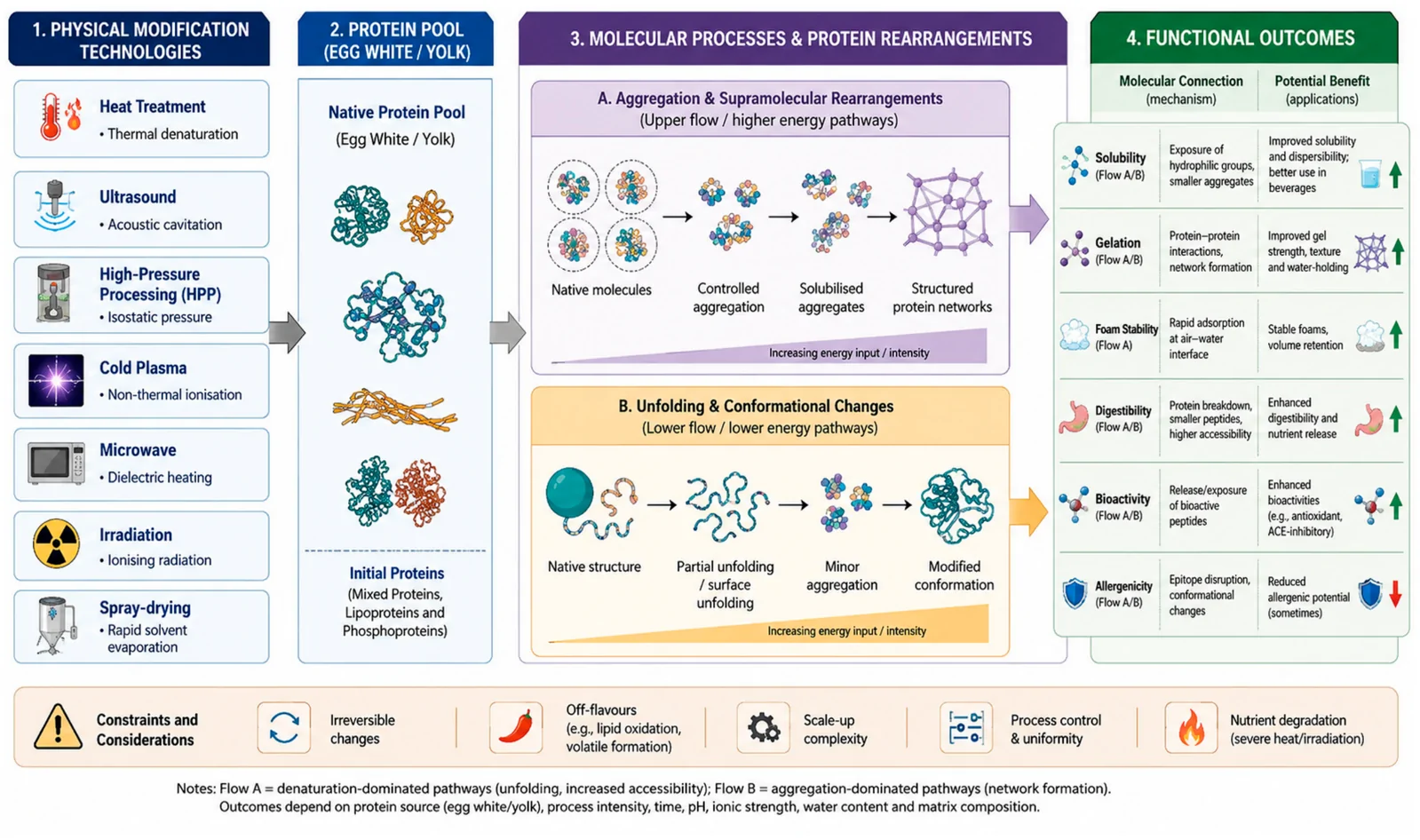
Physical modification technologies and protein rearrangements in egg-derived proteins. Schematic representation of major physical technologies, including heat treatment, ultrasound, high-pressure processing, cold plasma, microwave treatment, irradiation and spray-drying, and their effects on native egg white and egg yolk protein pools. The figure highlights two major molecular routes: aggregation and supramolecular rearrangement, and unfolding and conformational change. Functional outcomes include changes in solubility, gelation, foam stability, digestibility, bioactivity and allergenicity, while the main constraints include irreversible structural changes, off-flavor formation, scale-up complexity, process uniformity and nutrient degradation.

**Figure 4:**
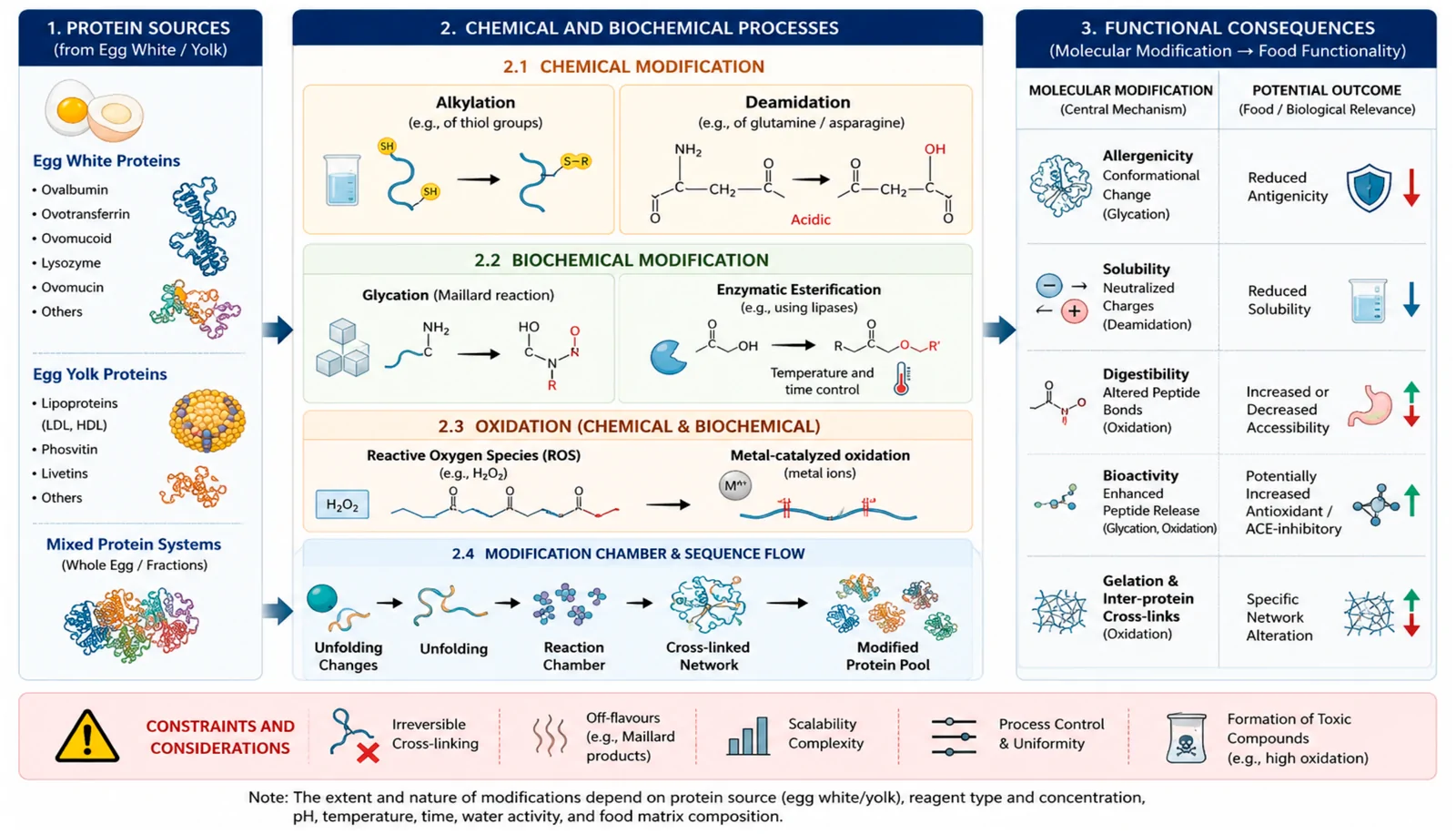
Chemical and biochemical modification of egg-derived proteins.

**Figure 5:**
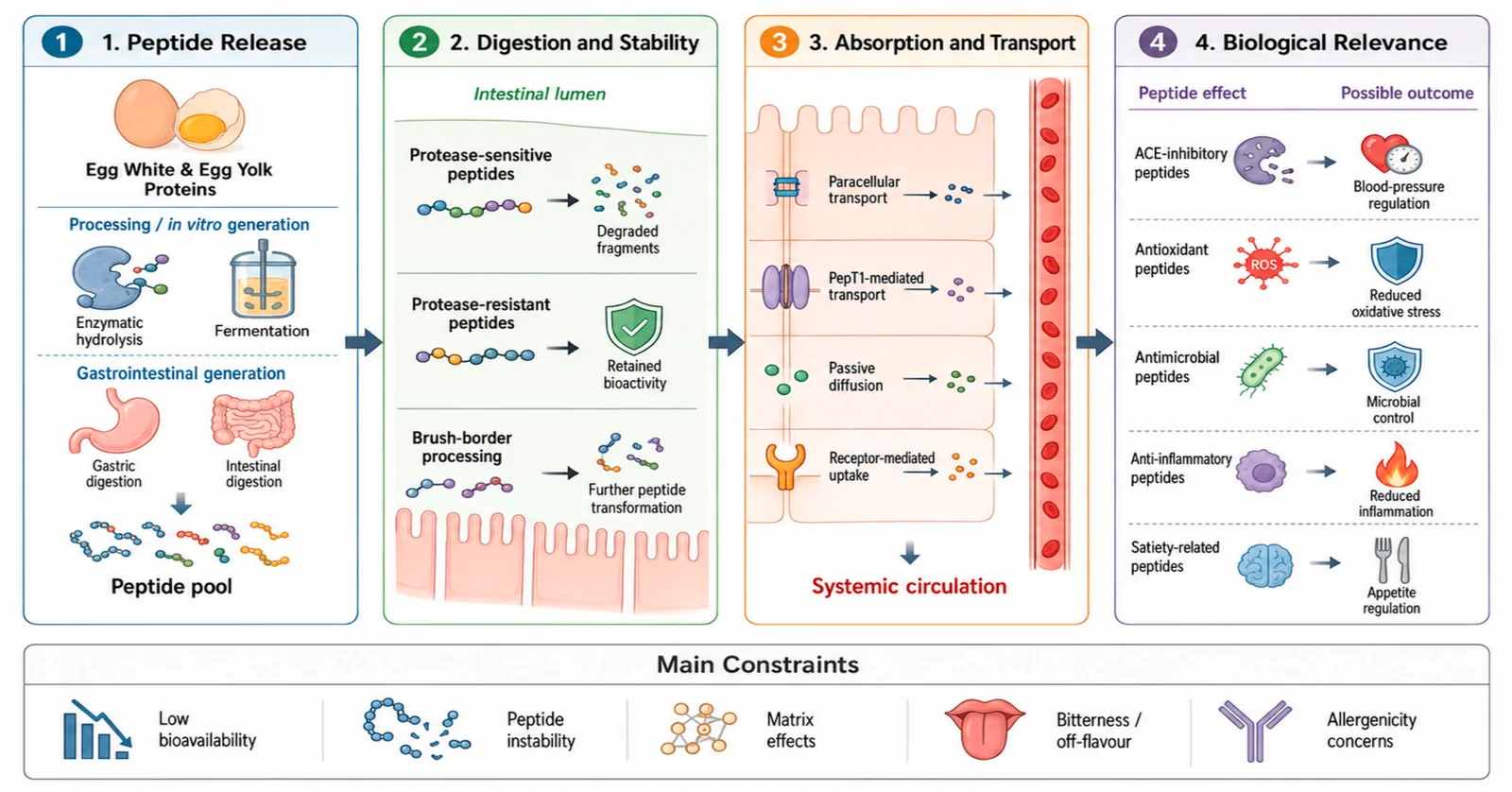
Egg-derived bioactive peptides: release, digestion, absorption and biological relevance.

**Table 1:** Main egg-derived proteins and their relevance for functional food design.

Egg Fraction	Main Protein or Protein Fraction	Main Structural or Biochemical Features	Techno-Functional Relevance	Biological or Nutritional Relevance	Main Limitations or Considerations	References
Egg white	Ovalbumin	Major egg white protein; heat-sensitive globular protein capable of unfolding and forming intermolecular interactions	Gelation, foaming, emulsion stabilization, film formation and structuring of heat-treated foods	Source of amino acids and precursor of peptides released through hydrolysis or gastrointestinal digestion	Functionality depends strongly on pH, temperature, ionic strength and interactions with other proteins	[1,2,6,52,53,54]
Egg white	Ovotransferrin (conalbumin)	Iron-binding glycoprotein sensitive to thermal and environmental conditions	Contributes to gel structure, interfacial behavior and protein-network formation	Antimicrobial activity associated partly with iron sequestration; precursor of bioactive peptides	Stability and functionality may change markedly during heating, pH adjustment or interactions with other proteins	[1,52,55]
Egg white	Ovomucoid	Glycoprotein and protease inhibitor with relatively high thermal and digestive stability	Limited direct technological role compared with ovalbumin or ovomucin	Protease-inhibitory activity and resistance to gastrointestinal digestion	Major egg allergen whose structural stability may contribute to persistent immunoreactivity after processing	[1,42,43,44,45,46,47,52]
Egg white	Lysozyme	Cationic enzyme that hydrolyzes peptidoglycan in susceptible bacterial cell walls	May participate in protein interactions and aggregation; relevant to preservation-oriented formulations	Natural antimicrobial activity and potential bioactive functionality	Application may be restricted by allergenicity and interactions with other food-matrix components	[1,52,55]
Egg white	Ovomucin	High-molecular-weight glycoprotein associated with the viscosity of thick albumen	Foam stabilization, viscosity modification, gel-like behavior and water retention	Potential precursor of peptides released following processing or digestion	Low solubility and complex extraction behavior may limit its use as an isolated ingredient	[1,2,52]
Egg white	Avidin	Biotin-binding glycoprotein	Limited direct role as a techno-functional food ingredient	Strong biotin-binding capacity	Raw or insufficiently heated avidin may reduce biotin availability; its nutritional relevance therefore depends on thermal processing	[1,52]
Egg yolk	Livetins	Water-soluble proteins located in the yolk plasma fraction	May contribute to interfacial behavior and interactions within food matrices	Nutritional protein fraction and potential precursor of bioactive peptides	Less extensively studied than egg white proteins and yolk lipoproteins; some livetins may have allergenic relevance	[1,56]
Egg yolk	Low-density lipoproteins	Lipid-rich protein assemblies located mainly in the yolk plasma fraction	Major contributors to emulsification and stabilization of oil–water interfaces	Carriers of lipid-associated nutrients and bioactive compounds	Sensitive to processing conditions that affect emulsion stability, protein structure and lipid oxidation	[1,3,5,13,56]
Egg yolk	High-density lipoproteins (lipovitellins)	Major protein components of yolk granules associated with phosvitin and mineral-mediated interactions	Contribute to structuring, emulsification, thermal aggregation and gel-like behavior	Source of amino acids and potential precursor of bioactive peptides	Solubility may be limited under certain conditions; functionality depends on granule disruption, ionic conditions and fractionation procedures	[1,4,5,56]
Egg yolk	Phosvitin	Highly phosphorylated protein with strong metal-binding capacity	May contribute to oxidative stability and emulsion behavior through interactions with metal ions, proteins and phospholipids	Mineral-binding activity; precursor of phosphopeptides with antioxidant and potential nutrient-delivery relevance	Extraction efficiency, cost, solubility and industrial scalability remain important barriers	[1,4,56,57]

**Table 2:** Enzymatic and fermentation-assisted strategies for modifying egg-derived proteins and their functional relevance.

Protein Source/Fraction	Modification Strategy	Main Structural or Biochemical Changes	Reported Functional or Biological Outcomes	Main Limitations/Critical Points	References
Egg white proteins and peptide fractions	Controlled proteolytic hydrolysis using neutral protease, Alcalase, pepsin, trypsin or other specified food-grade proteases	Peptide-bond cleavage, reduced molecular weight, altered surface hydrophobicity and generation of peptide fractions; batch-to-batch reproducibility was evaluated for Alcalase-generated peptides	Improved foaming capacity under limited neutral-protease hydrolysis; generation of peptide fractions with antioxidant, anti-inflammatory, ACE-inhibitory or antimicrobial potential; reproducible antioxidant activity and peptide sequences reported across Alcalase-treated batches	Extensive hydrolysis may reduce foam stability and gel strength, increase bitterness and generate heterogeneous peptide mixtures; biological evidence remains predominantly preclinical	[16,24,25,26,27,28,58]
Ovotransferrin/egg white hydrolysates	Proteolytic hydrolysis using thermolysin, thermoase or related enzymes	Release of ACE-inhibitory and other bioactive peptides	Antihypertensive potential reported in animal models; possible effects on oxidative stress and renin–angiotensin-related pathways	Human validation is limited; dose, peptide stability, absorption and matrix effects require further study	[24,55,59]
Egg yolk proteins	Protease- or lipase-assisted enzymatic treatment, including papain, lipase and flavor protease	Changes in secondary and tertiary structure, increased flexibility, altered sulfhydryl exposure and modified molecular interactions	Modification of gel texture, volatile binding and flavor profile; possible improvement of selected sensory or functional attributes	Effects depend strongly on enzyme type, treatment intensity and yolk fraction; excessive treatment may impair texture or flavor	[60,61]
Liquid egg yolk/yolk lipoproteins	Phospholipase A1 or A2 treatment	Disruption or rearrangement of lipoprotein micelles, altered phospholipid profile, increased protein solubility and modified interfacial behavior	Improved thermal stability, emulsifying capacity and oil–water interfacial performance	Requires careful process control; phospholipid modification may affect flavor, oxidation behavior, labeling and product-specific stability	[62]
Spray-dried egg yolk powde**r**	Lactic acid bacteria fermentation	Protein and lipid hydrolysis, smaller particle size, partial unfolding and changes in surface properties	Increased solubility, water dispersibility, dispersion stability and simulated gastrointestinal digestibility	Fermentation conditions must be optimized; microbial metabolites, acidification and flavor changes require control	[20,63]
Phosvitin and egg yolk phosphoproteins	Enzymatic hydrolysis or fermentation-assisted release of phosphopeptides	Release of phosphorylated peptides with metal-binding capacity	Potential mineral-binding, antioxidant and nutrient-delivery relevance	Extraction, purification, yield, cost and scalability remain important barriers	[28,57,64]
Ovalbumin and ovotransferrin	Transglutaminase-mediated cross-linking, often after heat or reducing pretreatment	Formation of covalent cross-links after exposure of reactive glutamine and lysine residues	Potential strengthening of gel networks, improved water retention and enhanced structuring capacity	Native globular proteins are relatively poor transglutaminase substrates unless partially unfolded; excessive cross-linking may reduce solubility	[65,66]
Mixed egg protein systems	Combined enzymatic and fermentation-assisted modification	Simultaneous proteolysis, structural rearrangement, metabolite formation and possible peptide release	Potential improvement of solubility, digestibility, bioactive potential and food-matrix compatibility	More difficult to standardize than purified systems; complex peptide mixtures complicate identification, reproducibility and regulatory evaluation	[20,27,28,63]

Abbreviation: ACE, angiotensin-converting enzyme.

**Table 3:** Physical modification technologies for egg-derived proteins: mechanisms, reported effects, functional relevance, potential applications and limitations.

Technology	Main Mechanism	Reported Effects on Egg-Derived Proteins	Functional Relevance and Potential Applications	Main Limitations	References
Thermal processing	Protein unfolding, exposure of hydrophobic and sulfhydryl groups, aggregation, disulfide exchange and gel-network formation	Formation of heat-induced networks; changes in texture, solubility, digestibility and peptide release, depending on treatment intensity	Gel formation and texture development → cooked egg products, bakery systems, desserts and other heat-structured foods	Excessive heating may produce compact aggregates, reduce solubility and foaming performance, and limit digestive-enzyme accessibility or peptide release	[34,35,41,67]
High-pressure processing	Disruption of non-covalent interactions, pressure-induced unfolding, aggregation and gelation	Modification of ovalbumin conformation and interfacial properties; pressure-induced gelation with changes in texture, syneresis and microstructure	Gelation, water retention and texture control → soft egg-protein gels, puddings and ready-to-eat protein-rich foods	High equipment cost; possible formation of overly dense networks; effects depend on pressure, holding time, temperature and product composition	[19,68]
Ultrasound treatment	Acoustic cavitation, shear forces, microstreaming, deaggregation, particle-size reduction and partial unfolding	Increased solubility, sulfhydryl exposure and surface hydrophobicity; improved foaming capacity and storage stability of pasteurized liquid egg white under controlled conditions	Improved interfacial adsorption and foaming → aerated foods and recovery of liquid egg-white functionality after pasteurization	Excessive sonication may cause oxidation and reaggregation, reducing solubility, foaming capacity, foam stability and emulsifying performance; scale-up uniformity remains difficult	[17]
Pulsed electric fields	Electric-field-induced changes in charge distribution, molecular conformation and non-covalent interactions	Potential modification of solubility, gelation, emulsification and foaming; direct evidence obtained with egg-derived proteins remains limited	Potential adjustment of solubility and interfacial behavior → mild processing of liquid egg ingredients; specific applications remain insufficiently demonstrated	Limited egg-specific evidence; outcomes depend on electric-field strength, pulse conditions, treatment-chamber design and product conductivity	[35,69]
Cold plasma	Reactive-species-induced oxidation, conformational rearrangement and changes in intermolecular interactions	Changes in solubility, foaming, emulsifying and gelling properties; possible microbial reduction under selected conditions	Modification of interfacial and gel-forming behavior, with potential microbial control → liquid egg white and other minimally heated egg ingredients	Excessive oxidation may damage amino acids or produce undesirable sensory changes; treatment uniformity, allergenicity and large-scale application require further assessment	[18]
Microwave treatment	Rapid volumetric dielectric heating and associated structural transitions	Controlled treatment can modify egg white protein conformation and improve the foaming properties of egg white powder	Faster structural modification and improved foam formation → egg white powders and aerated food products	Uneven temperature distribution may cause localized overheating, aggregation and loss of solubility	[70]
Irradiation	Ionizing-radiation-induced structural rearrangement, oxidation and changes in protein hydrophobicity	Selected doses increased protein hydrophobicity, foaming capacity and heat-set gelation in liquid egg white	Improved foaming and heat-induced network formation → aerated and gelled liquid egg-white products	Higher doses may promote oxidation and uncontrolled aggregation; regulatory requirements and consumer acceptance may restrict use	[71]
Spray-drying and other drying treatments	Dehydration, heat exposure, interfacial stress and powder formation	Changes in aggregation, hydrophobic-group exposure, rehydration, solubility, gelation and peptide stability; effects depend on drying method and process intensity	Production of stable and reconstitutable dry ingredients → egg white powder, whole-egg powder and egg-derived peptide preparations	Heat damage, oxidation, reduced solubility and loss of foaming or emulsifying performance; subsequent stability depends on packaging and storage conditions	[11,12,72,73]
Combined physical treatments (preheating–ultrasound , ultrasound–microwave and sequential ultrasound–heating)	Sequential control of unfolding, particle-size reduction, aggregation and interfacial rearrangement through complementary treatments	Improved foaming following preheating–ultrasound; formation of egg white microgels suitable for Pickering-emulsion stabilization following ultrasound–microwave treatment; improved foaming, emulsifying properties and in vitro digestibility following ultrasound–heating pretreatment	Controlled foaming, emulsification and particle formation → aerated products, egg white powders, protein microgels and Pickering-emulsion stabilizers	Benefits depend on treatment order, energy input, temperature and duration; combined treatments add process complexity and should be compared with the corresponding individual treatments	[74,75,76]

**Table 4:** Chemical and biochemical modification strategies for egg-derived proteins: mechanisms, reported relevance and translation constraints.

Modification Strategy	Protein Source or System	Main Molecular Mechanism	Reported Techno-Functional or Biological Relevance	Main Limitations and Translation Considerations	References
pH-shifting	Egg white proteins; egg white protein particles	Partial unfolding, charge modulation and exposure of hydrophobic and sulfhydryl groups	Improved emulsifying capacity, emulsion stability and heat stability	Extreme pH may cause irreversible aggregation, nutritional changes and sensory effects. Neutralization may increase salt content and water use; pH control and industrial reproducibility require evaluation	[15,77]
Maillard glycation with pullulan	Ovalbumin	Covalent attachment of pullulan to protein amino groups, producing steric stabilization and altered heat-induced transitions	Improved foaming stability and modified interfacial behavior	Reaction time, temperature and water activity require control. Excessive reaction may cause available-lysine loss, browning, flavor changes and formation of advanced Maillard products, including advanced glycation end products	[21]
Ultrasound-assisted Maillard glycation	Ovalbumin–xylose system	Ultrasound-assisted protein unfolding and accelerated covalent glycation	Improved foaming and emulsifying properties	Excessive sonication or glycation may cause oxidation, aggregation, non-uniform modification, lysine loss, browning and advanced Maillard-product formation	[78]
Dry-state glycation with maltodextrin	Egg white proteins	Covalent protein–carbohydrate conjugation and structural rearrangement under controlled temperature and humidity	Improved gel hardness, water-holding capacity, rheological behavior and gel microstructure	Temperature, humidity and reaction duration require strict control. Excessive reaction may cause nutritional losses, undesirable color or flavor and advanced Maillard products; scale-up requires uniform heat and moisture distribution	[79]
Maillard conjugation with citrus pectin	Ovalbumin	Formation of ovalbumin–pectin conjugates with changes in surface hydrophobicity and secondary and tertiary structure	Improved foaming capacity and foam stability	Protein-to-pectin ratio, reaction time, grafting degree and browning require control. Unreacted carbohydrate, lysine loss, sensory changes and performance in complete foods require evaluation	[80]
Maillard conjugation with dextran	Egg white proteins	Covalent protein–polysaccharide conjugation producing steric and interfacial stabilization	Improved emulsifying performance and colloidal stability	Outcomes depend on the protein-to-polysaccharide ratio, reaction conditions and degree of grafting. Residual carbohydrate, nutritional changes, browning, batch reproducibility and commercial-scale process control require assessment	[81]
Phosphorylation	Egg white powder; ovalbumin	Introduction of phosphate groups, increased negative charge, altered conformation and improved hydration	Improved solubility, foaming, emulsifying and water- and oil-holding properties; reduced IgG or IgE binding in selected in vitro systems	Phosphate reagent and concentration, degree of substitution, residual phosphate, salts and reaction by-products require control. Nutritional effects, total phosphorus content, clinical allergenicity, ingredient identity and regulatory status remain application-specific	[22,82]
Succinylation/acylation	Ovalbumin; egg white proteins	Introduction of negatively charged succinyl groups, increasing electrostatic repulsion and molecular flexibility	Improved solubility, interfacial behavior, foaming and emulsifying properties	Food-grade status of the acylating reagent, degree of substitution, residual reagent, reaction by-products and reaction termination require verification. Effects on amino acid availability, digestibility, ingredient identity, labeling and regulatory status must be considered	[23]
Succinylation of egg white hydrogels	Egg white proteins	Charge modification and restructuring of protein gel networks	Improved water-holding capacity, hardness, elasticity and hydrogel structure; reported mineral-binding capacity	Excessive substitution may alter texture, amino acid availability, digestibility and sensory quality. Residual reagents, purification requirements, regulatory status and performance in complete foods require evaluation	[83]
Succinylation of egg yolk LDL systems	Egg yolk low-density lipoproteins	Charge modification, reduced oxidation–aggregation and increased LDL-particle stability	Reduced off-flavor formation and improved thermal stability	Residual acylating reagent, reaction by-products, lipid oxidation, nutritional effects and food-grade processing require assessment. Regulatory classification and commercial-scale feasibility remain uncertain	[84]
Protein–polyphenol interactions and covalent conjugation	Egg white proteins; ovalbumin; lysozyme	Non-covalent association or covalent cross-linking through oxidized phenolic intermediates or free-radical-mediated reactions	Increased antioxidant potential; possible changes in interfacial behavior and IgE-binding properties	Polyphenol composition and oxidation state, conjugation yield, cross-linking degree, residual initiators or oxidants, unbound polyphenols and oxidation-derived products require control. Batch variability, amino acid availability, digestibility, color, flavor, astringency, ingredient identity and regulatory status must also be evaluated	[85,86]

Abbreviations: AGEs, advanced glycation end products; IgE, immunoglobulin E; IgG, immunoglobulin G; LDL, low-density lipoprotein.

**Table 5:** Biological activities and levels of evidence reported for egg-derived protein and peptide preparations.

Protein Source	Protein or Peptide Preparation	Production or Identification Approach	Reported Biological Activity	Evidence Level	Main Limitations or Critical Points	References
Egg white proteins	Mixed egg white protein hydrolysates and peptide fractions	Enzymatic hydrolysis, simulated gastrointestinal digestion, peptide fractionation, LC–MS/MS analysis and in silico screening	Antioxidant, anti-inflammatory, antimicrobial and ACE-inhibitory potential	Reviews, in vitro assays, cell models and selected animal studies	The reported activity depends on the parent protein, enzyme specificity, peptide size and sequence, digestion conditions and food-matrix composition; direct comparison among preparations remains difficult	[24,25,26,27,28,55]
Egg white proteins	Standardized peptide fractions obtained from enzymatic hydrolysates	Controlled enzymatic hydrolysis, peptide characterization and batch-stability assessment	Antioxidant potential and modulation of oxidative stress-related targets	In vitro assays, network pharmacology and molecular docking	Batch reproducibility, peptide composition and stability require standardized reporting before these fractions can be compared or used as functional ingredients	[58]
Egg white proteins	Hydrolysates evaluated in hypertension-related models	Proteolytic hydrolysis followed by oral administration in experimental animals	Blood-pressure reduction, vascular protection and modulation of oxidative stress- or renin–angiotensin-system-related pathways	Animal studies, including spontaneously hypertensive and DOCA-salt rat models	Translation to humans remains uncertain; dose, peptide bioavailability, digestion stability and food-matrix effects require further investigation	[59,89,90]
Egg protein	Dietary egg-protein hydrolysate preparation	Long-term dietary administration with vascular and metabolic endpoint assessment	Improvement of endothelial function	Randomized, double-blind, placebo-controlled human trial involving older adults with overweight or obesity	Human evidence is limited to a small number of preparations and study populations; product composition, dose–response relationships and generalizability require confirmation	[41]
Ovotransferrin-containing or ovotransferrin-depleted egg white proteins	Peptide-rich enzymatic hydrolysates	Enzymatic hydrolysis followed by preparation and evaluation of peptide-rich fractions	Antihypertensive potential and possible modulation of endothelial function, oxidative stress and vascular regulation	Predominantly animal evidence	The active peptide profiles, gastrointestinal stability, absorption and long-term biological effects remain insufficiently characterized	[55,59]
Egg white proteins	Antimicrobial peptide fractions obtained from hydrolysates	Enzymatic hydrolysis followed by antimicrobial screening and cell-related evaluation	Antimicrobial and anti-inflammatory activity against the acne-associated bacterium *Cutibacterium acnes* and the opportunistic skin pathogens *Staphylococcus aureus* and methicillin-resistant *S. aureus* (MRSA)	In vitro antimicrobial assays and cell-based evaluation	Efficacy in food systems has not been established; stability, effective concentration, safety, sensory effects and interactions with food-matrix components require assessment	[91]
Hen egg white proteins	Hydrolysate and selected peptide sequences	Peptidomic analysis, prediction of anti-inflammatory peptides, molecular docking and macrophage inflammation model	Reduction of inflammatory markers in lipopolysaccharide-stimulated macrophages	In vitro cell model, peptidomic analysis and molecular docking	Physiological relevance depends on gastrointestinal stability, absorption and the concentrations achievable in vivo	[92]
Defatted egg yolk proteins	Enzymatic hydrolysate and identified peptide fractions	Enzymatic hydrolysis, MALDI–TOF/MS peptide profiling and activity screening	Antioxidant and antimicrobial potential	In vitro assays and peptide identification	Evidence is limited mainly to experimental assays; peptide standardization, production scalability, stability and sensory effects require further study	[64]
Phosvitin and other yolk phosphoproteins	Phosphopeptide-rich hydrolysates	Enzymatic hydrolysis followed by the release and characterization of phosphopeptides	Mineral-binding, antioxidant and potential nutrient-delivery activity	Reviews and predominantly in vitro evidence	Extraction cost, solubility, purification yield, gastrointestinal stability and food-grade scalability remain important barriers	[57,64]
Protein-containing egg-processing fractions	Recovered protein hydrolysates and peptide-rich preparations	Protein recovery, enzymatic hydrolysis and bioactivity screening	Antioxidant, anti-inflammatory, metal-chelating and metabolic-protection potential	Review evidence and selected experimental studies	The composition of the recovered fractions and their mechanisms of action are not consistently characterized; bioaccessibility, clinical relevance and process scalability remain insufficiently established	[88]
Egg white proteins	Digested proteins, hydrolysates and derived peptides evaluated in intestinal systems	Simulated digestion and assessment in intestinal-barrier, inflammatory-response or gut-microbiota-related models	Potential modulation of intestinal barrier integrity, inflammation and microbiota-associated responses	Predominantly in vitro and animal evidence, supported by mechanistic reviews	Human evidence is scarce, and reported effects may depend on peptide composition, microbiota characteristics, digestion conditions, host physiology and food-matrix interactions	[28]

Abbreviations: ACE, angiotensin-converting enzyme; DOCA, deoxycorticosterone acetate; LC–MS/MS, liquid chromatography–tandem mass spectrometry; MALDI–TOF/MS, matrix-assisted laser desorption/ionization time-of-flight mass spectrometry; MRSA, methicillin-resistant *Staphylococcus aureus*.

**Table 6:** Factors influencing digestibility, bioaccessibility and bioavailability of egg-derived proteins and peptides.

Factor	Main Effect on Egg-Derived Proteins or Peptides	Functional or Nutritional Relevance	Main Limitation or Critical Consideration	References
Thermal processing	Protein unfolding, gel-network formation, aggregation and changes in enzyme accessibility	May improve structure and safety; may either increase or reduce digestibility, depending on treatment intensity	Excessive heating may generate compact networks and reduce peptide release	[34,35,67,93]
Cooking conditions and physiological model	Modification of gel structure, matrix hardness and digestion kinetics	Relevant for older adults and consumers with altered gastrointestinal conditions	Standard digestion models may not represent digestion in older adults, infants or individuals with compromised digestive function	[36,37]
Non-thermal processing	Conformational changes, partial unfolding, aggregation or changes in surface properties	May modify digestibility, solubility and functional performance	Effects depend strongly on intensity, matrix composition and technology	[17,18,19,34,35,69]
Peptide sequence and molecular size	Determines resistance to proteases, brush-border peptidases and intestinal uptake	Influences potential absorption and biological activity	Small size alone does not guarantee bioavailability or efficacy	[29,30,31,32,33,94]
Food matrix structure	Changes enzyme accessibility, water mobility, diffusion and peptide release	Critical in gels, emulsions, bakery systems, beverages and powders	Purified-protein results may not translate to complete foods	[30,31,32,67,95,96]
Lipid–protein interactions	Modify interfacial organization, oxidation behavior and digestion kinetics	Particularly relevant for egg yolk, whole egg and emulsion-based systems	Lipid composition and oxidation may affect functionality and bioactive compound delivery	[5,9,13,93]
Packaging and storage of powdered eggs	May influence moisture, oxidation, solubility, caking, composition and thermal stability	Relevant for industrial egg powders and dry functional ingredients	Stored powders may behave differently from freshly prepared laboratory dispersions	[11,12]
In vitro digestion model	Allows controlled screening of hydrolysis, peptide release and bioaccessibility	Useful for comparing processing and matrix effects	Does not fully reproduce absorption, microbiota, hormonal regulation or inter-individual variability	[31,32]
Intestinal transport	Determines whether intact peptides or fragments cross the intestinal barrier	Essential for systemic biological relevance	Evidence remains limited; animal or cell models cannot be directly generalized to humans	[29,33,94]

**Table 7:** Application-specific performance and validation requirements for egg-derived proteins in food systems.

**Application Area**	**Relevant Egg-Derived Ingredient**	**Application-Specific Technological Role**	**Product-Relevant Performance Criteria**	**Sensory, Storage and Industrial Considerations**	**Current Evidence and Validation Needs**	**References**
Aerated bakery and confectionery products	Egg white proteins, particularly ovalbumin, ovomucin and mixed albumen proteins	Foam formation and stabilization, volume development and structure setting during heating	Batter or foam stability, bubble-size distribution, baked volume, crumb or cellular structure, moisture retention and fracture behavior	Sugar, fat and salt may alter aeration; excessive treatment may reduce foam stability or produce undesirable color, flavor or texture	Foaming has been extensively evaluated in protein systems, but modified ingredients require further validation in complete bakery and confectionery formulations during processing and storage	[2,6,16,17,70,97]
Gelled foods, desserts and structured matrices	Egg white proteins, ovalbumin-rich fractions and mixed egg protein gels	Gel formation, water retention, texture development and matrix structuring	Gel strength, elasticity, syneresis, water-holding capacity, microstructure, digestibility and stability during heating and storage	Excessive firmness or cross-linking may reduce solubility, sensory acceptability or digestive-enzyme accessibility; texture requirements differ among desserts, soft foods and protein-enriched products	Gelation is well documented in model systems, whereas product-specific evidence combining texture, sensory quality, digestion and shelf-life remains more limited	[6,34,35,36,65,66,68,79,83]
Sauces, dressings and other emulsion-based foods	Whole egg yolk, low-density lipoproteins, phospholipids and phosvitin-containing yolk fractions	Oil–water emulsification, droplet stabilization, viscosity control and lipid structuring	Droplet-size distribution, creaming resistance, rheological behavior, thermal and acid stability, and resistance to coalescence	Lipid oxidation, off-flavor development, color changes and loss of emulsifying performance during storage must be assessed alongside physical stability	Egg yolk is established as an emulsifying ingredient, but modified yolk preparations require validation under product-relevant oil content, pH, salt concentration, homogenization, heating and storage conditions	[3,5,9,56,62,84,98,99]
Powdered egg ingredients and dry food systems	Whole egg powder, egg white powder, egg yolk powder and egg-derived peptide powders	Shelf-stable protein supply and provision of foaming, emulsifying or gel-forming properties after reconstitution	Rehydration rate, dispersibility, solubility, powder flow, caking tendency and recovery of foaming, emulsifying or gelation performance	Oxidation, off-flavors, color changes and storage-related loss of functionality depend on drying conditions, packaging and storage environment	Industrial use is established, but comparative studies should evaluate functionality after realistic storage and reconstitution rather than only immediately after drying	[11,12,63,72,73,98,100]
Protein-enriched beverages and liquid foods	Egg white protein hydrolysates, soluble egg white fractions and modified protein dispersions	Protein enrichment, dispersion stability and delivery of peptide-containing ingredients	Solubility, turbidity, sedimentation, viscosity, pH stability and resistance to aggregation during pasteurization and storage	Bitterness, egg-derived flavor, color, mouthfeel, allergen declaration and consumer acceptance may limit incorporation levels	Much of the available evidence concerns solubility or peptide activity in model systems; complete beverage formulations require sensory, processing, shelf-life and bioavailability assessment	[15,16,24,25,26,27,28,29,30,31,32,33,49,58,81,94]
Edible films and coatings	Egg white proteins and edible protein–polysaccharide or protein–polyphenol composites	Formation of consumable surface layers with mechanical, gas-barrier, moisture-barrier or active properties	Film continuity, adhesion, tensile behavior, flexibility, oxygen and water-vapor permeability, and antimicrobial or antioxidant performance on foods	Taste, odor, appearance, mouthfeel, moisture sensitivity, storage stability and mandatory management of egg-allergen information must be considered	Material-level results should be complemented by studies on coated foods, including sensory acceptability, microbial quality and shelf-life	[85,86,101,102]
Active packaging and other food-contact materials	Egg-protein-containing films, composites or active layers not intended for consumption	Structural or active component of packaging systems, including potential antioxidant or antimicrobial action	Mechanical integrity, barrier performance, active-compound stability, migration and activity throughout the intended contact period	These materials require food-contact safety assessment, migration evaluation and allergen-risk management distinct from those applicable to edible materials	Evidence obtained from film characterization alone is insufficient; testing should include migration, food-contact compatibility, packaged-product stability and compliance with the applicable jurisdiction	[85,86,101,102]
Delivery systems for bioactive compounds and nutraceutical ingredients	Egg white protein gels and assemblies, egg yolk lipoproteins, lecithin-containing systems and peptide-rich fractions	Encapsulation, protection and release of lipophilic or hydrophilic compounds	Encapsulation efficiency, physical stability, retention during processing and storage, release kinetics, bioaccessibility and intestinal transport	Matrix compatibility, flavor, color, allergenicity, ingredient standardization and stability during food processing may restrict application	Many systems have been evaluated in simplified dispersions or digestion models; their performance must be confirmed in complete foods and under realistic storage and gastrointestinal conditions	[6,7,8,9,24,25,26,27,28,29,30,31,32,33,57,81]
Meat, seafood and other structured food systems	Egg white proteins, egg white gels, hydrolysates and cross-linked protein networks	Binding, water retention, texture reinforcement and stabilization of composite protein structures	Cooking yield, water-holding capacity, texture, structural integrity and stability under salt and thermal processing conditions	Interactions with muscle proteins, fat and salt may affect texture, flavor, appearance and allergen management	The technological potential is supported mainly by protein-network and gelation evidence; direct validation in specific meat or seafood products should be distinguished from results obtained in model systems	[6,34,65,66,83]

**Table 8:** Safety, allergenicity, sensory and regulatory considerations for modified egg-derived proteins.

Critical Aspect	Relevance for Egg-Derived Proteins	Main Risk or Limitation	Recommended Evaluation Approach	References
Egg allergenicity	Egg white and yolk contain clinically relevant allergens, including ovomucoid, ovalbumin, ovotransferrin, lysozyme and yolk allergens	Processing cannot be assumed to eliminate allergenic potential; changes in IgE binding are protein- and process-dependent	Combine protein profiling, IgE-binding assays, digestion models and, where justified, in vivo or clinical assessment	[42,43,44,45,46,47,103,104,105,106]
Processing-dependent epitope changes	Heat, hydrolysis, glycation, ultrasound, high pressure, irradiation and cold plasma may alter conformational and linear epitopes	The same treatment may mask, destroy or expose epitopes according to treatment intensity, allergen structure and food matrix	Evaluate allergenicity before and after processing and simulated gastrointestinal digestion	[42,43,44,45,46,47,103,104,105]
Ovomucoid stability	Ovomucoid is relatively resistant to heating and proteolysis and is associated with persistent egg allergy	Structural modification may either mask or expose relevant epitopes	Use allergen-specific assessment rather than total-protein immunoreactivity alone	[42,43,44,45,46,47,104]
Allergen detection in processed foods	Modified foods may contain several egg allergens that differ in their stability, extractability and detectability	Reliance on a single marker may misrepresent residual allergen content	Use validated complementary methods, including multi-allergen LC–MS/MS analysis when appropriate	[47,106]
Hydrolysate bitterness and off-flavors	Hydrolysis, fermentation, oxidation and storage may generate bitter peptides or undesirable volatile compounds	Sensory defects may restrict ingredient incorporation and consumer acceptance	Combine sensory analysis with peptide profiling, oxidation markers and appropriate mitigation strategies	[49,107,108]
Oxidative and storage deterioration	Yolk fractions and egg powders are susceptible to lipid and protein oxidation	Oxidation may impair flavor, color, solubility, functionality and nutritional quality	Monitor primary and secondary oxidation products, volatile markers and functional performance throughout shelf-life	[11,12,84,98]
Chemical modification and ingredient acceptability	Succinylation, phosphorylation and selected conjugation reactions may improve specific properties	Reagent use, reaction products, residual compounds and ingredient identity may restrict food application	Verify food-grade status, control residual compounds and determine the applicable ingredient designation	[22,23,82,83,84,85,86]
Maillard reaction products	Controlled glycation may modify functionality and immunoreactivity	Excessive reaction may cause browning, lysine loss, off-flavors or unwanted reaction products	Control time, temperature, water activity and carbohydrate type and assess nutritional and sensory consequences	[21,78,79,80,81,104]
Population-specific applications	Egg-derived ingredients may be used in foods intended for older adults, individuals with dysphagia or consumers with specific nutritional requirements	Digestibility, texture, swallowing safety, allergenicity and claim substantiation may require targeted assessment	Apply product- and population-relevant digestion, texture, safety and sensory models	[36,37,38,39,40,41,103]
Allergen labeling and claims	Egg-derived ingredients are subject to allergen-declaration requirements in many jurisdictions	Modification, hydrolysis or encapsulation does not automatically remove labeling obligations	Confirm ingredient identity, allergen declaration, processing description and compliance of consumer-facing claims	[48,103,106]

Abbreviations: IgE, immunoglobulin E; LC–MS/MS, liquid chromatography–tandem mass spectrometry. Gal d 1–6 denotes the standardized nomenclature used for the principal hen’s egg allergens included in multi-allergen analyses.

**Table 9:** Key barriers and future research priorities for the industrial translation of egg-derived functional ingredients.

Development Area	Current Limitation	Why it Matters for Industrial Translation	Recommended Future Direction	References
Process scalability	Many studies remain at laboratory scale using purified proteins or simplified dispersions	Industrial systems involve larger volumes, variable raw materials, different heat and mass transfer conditions, and stricter reproducibility requirements	Validate modification technologies at pilot scale and report process parameters relevant to industrial implementation	[17,18,19,20,62,68,72,100,109]
Peptide standardization	Hydrolysates often contain complex and variable peptide mixtures	Batch variability limits comparison between studies, quality control, bioactivity attribution and regulatory substantiation	Combine controlled hydrolysis with LC–MS/MS profiling, peptide mapping, molecular-weight distribution and batch-to-batch validation	[58,88,109,110,111]
Fractionation and purification	Bioactive peptides are difficult to separate without losses in yield, activity or cost efficiency	Industrial production requires scalable, food-grade and economically feasible separation processes	Develop mild membrane, chromatographic or hybrid fractionation strategies that preserve bioactivity while reducing cost	[58,88,109,111]
Food-grade modification	Some chemical modifications require reagents or reaction conditions that may limit consumer or regulatory acceptability	Clean-label expectations and ingredient identity influence product adoption	Prioritize enzymatic, fermentation-assisted or mild physical strategies; when chemical modification is used, control residual reagents and labeling implications	[20,21,22,23,75,76,77,78,79,80,81,82,83,84,85,86,103,112]
Sensory quality	Hydrolysis, fermentation, oxidation and Maillard reactions may generate bitterness, off-flavors or color changes	Sensory defects can prevent product acceptance even when functionality or bioactivity is promising	Integrate sensory evaluation, volatile analysis, bitterness prediction and mitigation strategies early in development	[49,84,98,107,108,109]
Storage and powder stability	Egg powders and peptide powders may lose solubility, functionality or oxidative stability during storage	Industrial ingredients may be stored for months before incorporation into foods	Evaluate drying, packaging, water activity, oxidation, caking, rehydration and functionality during realistic shelf-life conditions	[11,12,72,73,98,100]
Allergenicity and safety	Processing may reduce, mask, expose or generate allergenic structures	Egg-derived ingredients remain allergen-containing materials and require careful risk assessment	Combine allergen quantification, IgE-binding assays, simulated digestion and, where relevant, in vivo or clinical evidence	[42,43,44,45,46,47,48,103,104,105,106]
Bioavailability and evidence level	Many biological effects are based on in vitro assays or animal models	Functional food claims require stronger evidence than biochemical activity alone	Link peptide identification with digestion stability, intestinal transport, systemic availability and human intervention studies	[29,30,31,32,33,41,95,110,112]
Real food matrix validation	Modified proteins are often tested in model systems rather than commercial-type foods	Matrix composition can alter functionality, digestion, sensory properties and storage stability	Test modified ingredients in application-specific matrices such as foams, gels, emulsions, powders, beverages and bakery systems	[6,7,8,9,17,63,67,68,98,99,100,101,102]
Sustainability and by-product valorization	Egg-processing fractions and by-products remain underutilized in high-value applications	Valorization can reduce waste and improve resource efficiency	Develop integrated valorization chains for salted egg white, defatted yolk protein, eggshell membrane and other egg-processing streams	[88,98,100,112]

Abbreviations: IgE, immunoglobulin E; LC–MS/MS, liquid chromatography–tandem mass spectrometry.

## Data Availability

No new data were created or analyzed in this study. Data sharing is not applicable to this article.
